# *Pseudomonas aeruginosa* uses a cyclic-di-GMP-regulated adhesin to reinforce the biofilm extracellular matrix

**DOI:** 10.1111/j.1365-2958.2009.06991.x

**Published:** 2010-01-17

**Authors:** Bradley R Borlee, Aaron D Goldman, Keiji Murakami, Ram Samudrala, Daniel J Wozniak, Matthew R Parsek

**Affiliations:** 1 Department of Microbiology, University of WashingtonBox 357242, Seattle, WA, 98195-7242, USA; 2 Infectious Disease, Microbiology, Center for Microbial Interface Biology, The Ohio State University460 West 12th Avenue, Columbus, OH, 43210, USA

## Abstract

*Pseudomonas aeruginosa*, the principal pathogen of cystic fibrosis patients, forms antibiotic-resistant biofilms promoting chronic colonization of the airways. The extracellular (EPS) matrix is a crucial component of biofilms that provides the community multiple benefits. Recent work suggests that the secondary messenger, cyclic-di-GMP, promotes biofilm formation. An analysis of factors specifically expressed in *P. aeruginosa* under conditions of elevated c-di-GMP, revealed functions involved in the production and maintenance of the biofilm extracellular matrix. We have characterized one of these components, encoded by the PA4625 gene, as a putative adhesin and designated it *cdrA*. CdrA shares structural similarities to extracellular adhesins that belong to two-partner secretion systems. The *cdrA* gene is in a two gene operon that also encodes a putative outer membrane transporter, CdrB. The *cdrA* gene encodes a 220 KDa protein that is predicted to be rod-shaped protein harbouring a β-helix structural motif. Western analysis indicates that the CdrA is produced as a 220 kDa proprotein and processed to 150 kDa before secretion into the extracellular medium. We demonstrated that *cdrAB* expression is minimal in liquid culture, but is elevated in biofilm cultures. CdrAB expression was found to promote biofilm formation and auto-aggregation in liquid culture. Aggregation mediated by CdrA is dependent on the Psl polysaccharide and can be disrupted by adding mannose, a key structural component of Psl. Immunoprecipitation of Psl present in culture supernatants resulted in co-immunoprecipitation of CdrA, providing additional evidence that CdrA directly binds to Psl. A mutation in *cdrA* caused a decrease in biofilm biomass and resulted in the formation of biofilms exhibiting decreased structural integrity. Psl-specific lectin staining suggests that CdrA either cross-links Psl polysaccharide polymers and/or tethers Psl to the cells, resulting in increased biofilm structural stability. Thus, this study identifies a key protein structural component of the *P. aeruginosa* EPS matrix.

## Introduction

Biofilm bacteria are embedded in a self-produced extracellular matrix. This matrix is thought to provide multiple functions for the community, serving as a scaffold holding biofilm cells together and providing protection from some antimicrobials. Despite the importance of the EPS matrix, we know surprisingly little about it. Although the exact composition is uncertain, extracellular polysaccharides, protein and nucleic acids have each been shown to be present. *Pseudomonas aeruginosa* is a model organism for the study of biofilms. Early studies determined that the *P. aeruginosa* extracellular matrix was comprised mostly of a mannose-rich polysaccharide, with some DNA, RNA and protein (Eagon, 1962). Subsequently, extracellular DNA was identified as an important part of the matrix (Whitchurch *et al.*, 2002), with early stage biofilms being susceptible to exogenous DNase treatment. The Pel and Psl polysaccharides have also been shown to be important components of the matrix (Friedman and Kolter, 2004a,b;, Jackson *et al.*, 2004; Matsukawa and Greenberg, 2004). The Pel polysaccharide is a glucose-rich polymer that primarily plays a role after surface attachment (Friedman and Kolter, 2004a; Vasseur *et al.*, 2005). Psl has been characterized as mannose-rich (Friedman and Kolter, 2004b; Ma *et al.*, 2007) and is important for attachment to abiotic and biotic surfaces relevant to Cystic Fibrosis (CF) infections such as mucin and airway epithelial cells. It also has an important role maintaining biofilm structure post-attachment (Ma *et al.*, 2006).

Understanding the regulation of matrix component expression is an area of intense interest. Recent data suggest that cyclic diguanylate (c-di-GMP) positively modulates production of matrix components at the transcriptional and allosteric level for *P. aeruginosa* and other Gram-negative species (Simm *et al.*, 2004; Tischler and Camilli, 2004; Hickman *et al.*, 2005; Gjermansen *et al.*, 2006; Thormann *et al.*, 2006). The emerging paradigm is that high intracellular levels of c-di-GMP promote the biofilm lifestyle through matrix production, while lower levels of c-di-GMP promote motility and the planktonic lifestyle.

A clear manifestation of this principle is seen in the rugose small colony variants (RSCVs) of *P. aeruginosa* that emerge from laboratory grown biofilms and chronic CF airway infections. RSCVs are characterized by hyper-biofilm formation and increased antimicrobial resistance compared with the wild-type ancestral strains from which they are derived (Haussler *et al.*, 1999; Drenkard and Ausubel, 2002; Boles *et al.*, 2004; Kirisits *et al.*, 2005). These attributes probably contribute to the ability of RSCVs to persist in the host environment, which is supported by the work of Smith *et al.* who demonstrated that mutations that confer the phenotype are actively selected for in the CF airways (Smith *et al.*, 2006). One genetic route to RSCV formation is a mutation in *wspF,* which results in activation of the diguanylate synthase, WspR (D'Argenio *et al.*, 2002; Hickman *et al.*, 2005). Thus, RSCVs are also characterized by elevated intracellular levels of c-di-GMP. At least two important matrix components, Pel and Psl, are overproduced in these variants as a consequence, and both contribute to the RSCV phenotype (Starkey *et al.*, 2009).

Starkey *et al.* recently identified the genes transcriptionally induced in response to elevated c-di-GMP in *P. aeruginosa* (Starkey *et al.*, 2009). The number of genes upregulated was surprisingly small (35), with a majority belonging to the *pel* and *psl* gene clusters. We hypothesized that in addition to the *pel* and *psl* clusters, c-di-GMP induced genes may encode functions that also contribute to EPS matrix production. In this manuscript we demonstrate that one of the genes identified in the Starkey study, PA4625, appears to encode an important protein component of the EPS matrix. PA4625 and PA4624 are predicted to comprise a two-partner secretion (TPS) system encoding a large secreted adhesin, and its transporter. We have designated PA4625 and PA4624 as *cdrA* (cyclic diguanylate-regulated TPS partner A) and *cdrB* (cyclic diguanylate-regulated TPS partner B) respectively, based on their regulation by c-di-GMP and their similarity to TPS proteins. Computational analysis predicts that CdrA is a long, rod-shaped protein containing a β-helix structural motif.

CdrA shares many similar features with proteins from the autotransporter and TPS systems. The prototypical TPS system is the filamentous haemagglutinin (FHA) and its FhaC secretion partner from *Bordetella pertussis*. FHA is an adhesin with multiple binding sites and activities which include binding to cilia in a galactose-inhibitible manner (Tuomanen *et al.*, 1988), integrin recognition required for binding to macrophages (Relman *et al.*, 1990), aggregation that is abolished by the addition of cyclodextrin (Menozzi *et al.*, 1994a), and binding to laryngeal epithelial cells (van den Berg *et al.*, 1999). FHA is responsible for *B. pertussis* growth as aggregates within the alveolar lumen of murine lungs and these clusters are similar to the aggregates that are observed when *B. pertussis* is cultivated *in vitro* (Menozzi *et al.*, 1994a). FHA is a secreted protein that is 500 Angstroms in length and predicted to form a rod-shaped molecule with a beta-helical structure (Kajava *et al.*, 2001). FHA is secreted by FhaC which shares similarity with outer membrane proteins involved in the export and activation of haemolysins (Willems *et al.*, 1994). Another β-helix protein that has been implicated in biofilm formation is Antigen 43 in *Escherichia coli*. This protein is also a large secreted adhesin that promotes auto-aggregation and biofilm formation (Henderson *et al.*, 1997; Hasman *et al.*, 1999; Danese *et al.*, 2000; Kjaergaard *et al.*, 2000). Antigen 43 has also been implicated in pathogenesis and binding to eukaryotic cells (Ulett *et al.*, 2007; de Luna *et al.*, 2008).

In this study, we show that CdrA is expressed in response to high c-di-GMP and in biofilm cultures. We show that CdrA is secreted outside the cell and binds to the Psl polysaccharide promoting auto-aggregation in liquid culture and biofilm formation on surfaces. Collectively, our data indicate that CdrA contributes to biofilm structural integrity by maintaining Psl association with the biofilm community.

## Results

### Computational analyses predict that PA4625 encodes a filamentous β-helical protein secreted by its transporter, encoded by PA4624

**Fig. 1:**
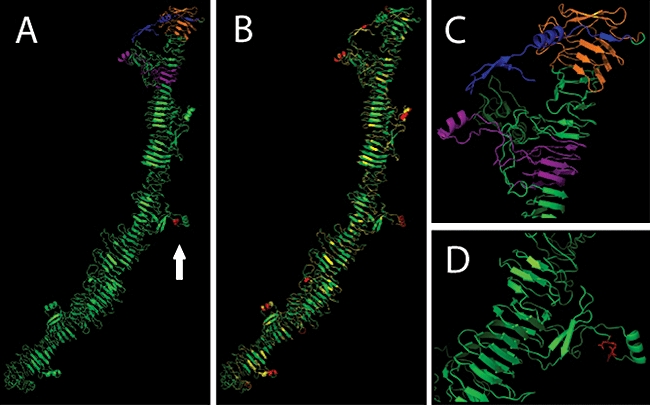
A diagram of a tertiary structure model of CdrA. The model was constructed by the *de novo* modelling of six separate segments and reattaching these segments by overlapping regions of structures. A. The global model of CdrA predicts a β-helix dominated structure with several exposed loops containing α-helices. The arrow indicates a predicted integrin binding motif. B. The same model was colour-coded to represent a trinary confidence measure based on secondary structure. Green indicates high confidence, yellow indicates intermediate confidence and red indicates low confidence. C. The N-terminal domain of CdrA contains a predicted signal peptide (blue), a haemagglutination site (orange) and a putative sugar-binding domain (purple). D. An exposed loop of the CdrA model contains a predicted integrin binding motif (red). Amino acids of the integrin binding motif are highlighted in red.

The Pseudomonas genome website has annotated PA4625 and PA4624 as hypothetical proteins, putatively encoded by a two gene operon. To gain insight into potential functions for PA4625 and PA4624, we performed a bioinformatics analysis. A Phyre analysis was conducted to identify remote homology to known structures (Bennett-Lovsey *et al.*, 2008). Phyre revealed that PA4625 and PA4624 are most likely members of a TPS. The PA4625 gene is predicted to encode a 220 kDa protein with a type I export signal (shaded in blue, Fig. 1C). The N-terminal region was most similar to the secretion domains of the large adhesins, FHA and HMW1 of *B. pertussis* and *Haemophilus influenzae* respectively. PA4624 is most similar to a membrane protein, FhaC, which is a member of *omp85*/*tpsB* transporter family. Based upon these homologies, and the fact that their expression was controlled by c-di-GMP, we renamed PA4625 and PA4624 as *cdrA* (cyclic diguanylate-regulated TPS partner A) and *cdrB* (cyclic diguanylate-regulated TPS partner B).

The secondary structure of CdrA was predicted by PsiPred (Jones, 1999; Bryson *et al.*, 2005) to be dominated almost entirely by β-strands (see Fig. S1). Given this result and the size of the protein, we predict that CdrA has a beta helix tertiary structure (Fig. 1A). The tertiary structure was modelled by an iterative approach in which segments of the protein were modelled *de novo* and reattached via structural alignment of overlapping regions (presented in Fig. 1). The secondary structure and function of CdrB was predicted by I-TASSER (Zhang, 2008) to be most similar to FhaC which forms a β-barrel channel in the outer membrane for the outward translocation of FHA. The highest-ranking tertiary structure of CdrB as predicted by I-TASSER is presented in Fig. S2.

A search of the Conserved Domains Database (Marchler-Bauer *et al.*, 2007) revealed that amino acids 45–153 of CdrA contain a carbohydrate-dependent haemagglutination activity domain (orange shading, Fig. 1A and C). These domains are found in secreted proteins which include haemagglutinins, haemolysins and members of the TPS secretion family. In addition, a sugar-binding domain harbouring a glycine-rich motif (PS50315) spanning amino acids 332–477 was predicted by PROSITE and PDBeMotif 1.0 (purple shading, Fig. 1A and C). A 42 amino acid signal peptide sequence (shaded blue, Fig. 1A and C) was predicted by SignalP 3.0 (Bendtsen *et al.*, 2004) to be cleaved between residues A42 and A43 of CdrA (*P* = 0.993). In other TPS secretion systems, similar signal peptides have been shown to be important for Sec-dependent secretion across the inner membrane (Chevalier *et al.*, 2004). Analysis of the primary amino acid sequence also identified an Arg-Gly-Asp (RGD) sequence motif at amino acids 1019–102, which may function as an integrin recognition site similar to eukaryotic extracellular matrix proteins (arrow in Fig. 1A and red shading, Fig. 1D).

### Verification that *cdrA* transcription is c-di-GMP-dependent and that *cdrAB* constitutes an operon

**Fig. 2:**
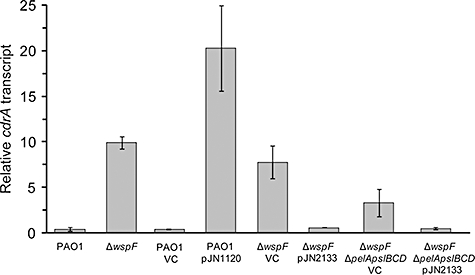
RT-PCR data of *cdrA* expression under conditions of high and low c-di-GMP. *cdrA* is highly expressed under conditions where c-di-GMP is elevated. Relative transcript levels for *cdrA* are shown for wild-type *P. aeruginosa* (PAO1), a *wspF* mutant (elevated c-di-GMP), PAO1 expressing a diguanylate cyclase, PA1120 (elevated c-di-GMP), a *wspF* mutant expressing a c-di-GMP degrading phosphodiesterase PA2133 (low c-di-GMP), a *wspFpelApslBCD* mutant harbouring a vector control (elevated c-di-GMP) and a *wspFpelApslBCD* mutant expressing a c-di-GMP degrading phosphodiesterase (low c-di-GMP). Data are shown for four biological replicates.

Previous transcriptional profiling studies suggested that *cdrAB* expression is elevated under conditions of high intracellular c-di-GMP (Hickman *et al.*, 2005; Hickman and Harwood, 2008; Starkey *et al.*, 2009). To verify these results, we measured *cdrA* transcript levels in the wild-type and a *wspF* mutant strain (high c-di-GMP). The levels of relative *cdrA* transcript were 27-fold higher in the *wspF* mutant strain (Fig. 2).

To verify that *cdrAB* expression is controlled by c-di-GMP and not a side-effect of auto-aggregation seen in the *wspF* background, we engineered a *wspFpelApslBCD* triple mutant strain that retains elevated c-di-GMP, but does not auto-aggregate (Starkey *et al.*, 2009). First we verified that *wspFpelApslBCD* displayed elevated levels of *cdrA* expression compared with the parental PAO1 strain (Fig. 2). Next we examined the effect of c-di-GMP depletion on *cdrA* expression. Depletion of c-di-GMP was achieved through expression of a c-di-GMP phosphodiesterase encoded by the PA2133 gene (Hickman *et al.*, 2005). Under conditions where c-di-GMP has been depleted in the *wspFpelApslBCD* mutant, *cdrA* transcripts were 7-fold lower than the *wspFpelApslBCD* strain harbouring the vector control (Fig. 2).

The Operon Finding Software v2.1 of the Pseudomonas genome website predicts that *cdrA* and *cdrB* are part of a two gene operon. To test this, we designed primers to be used in reverse transcription polymerase chain reaction (RT-PCR) that are internal to the *cdrA* and *cdrB* open reading frames. Only a dicistronic mRNA containing coding sequence of both ORFs would produce an amplicon. We tested mRNA harvested from both a PAO1 and a PAO1*wspF* mutant background. Both PAO1 and PAO1*wspF* strains produced a DNA band of the correct size (see Fig. S3), indicating that *cdrA* and *cdrB* are transcribed as part of the same transcript.

### CdrA appears to be processed, and is found associated with cells as well as in culture supernatants

**Fig. 3:**
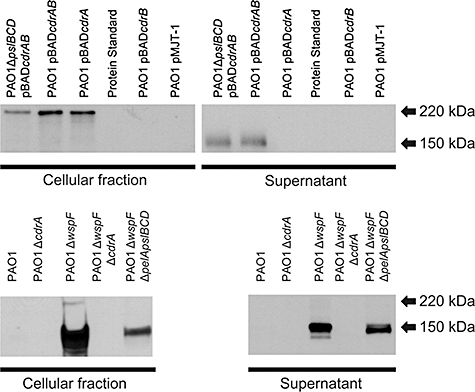
Western blot analysis of CdrA. Polyclonal antisera was used to monitor CdrA expression. In the top panel, CdrA was detected in the cellular fractions and culture supernatants in strains engineered to overexpress CdrA. The detection of CdrA in supernatants was dependent upon CdrB. In the lower two panels, CdrA expression was only detected in planktonic cultures of a *ΔwspF* background, not PAO1.

CdrA sequence analysis predicts that it is secreted or cell-surface associated. To determine CdrA localization and whether it is post-translationally processed like other TPS adhesins (e.g. FHA), we generated polyclonal antisera to a fragment of CdrA. Western analysis indicates that two primary forms of CdrA are produced, an unprocessed version of the protein (∼220 kDa) and a smaller, processed version (∼150 kDa) (Fig. 3). Primarily full-length CdrA appears to be cell-associated, while only the processed version is present in culture supernatants (Fig. 3A). This is consistent with previous reports of FHA, which is also both cell surface-associated and secreted into the extracellular environment (Mazar and Cotter, 2007). No CdrA was seen in the supernatant of the strain overexpressing only *cdrA*, presumably because of the absence of CdrB to transport CdrA across the outer membrane. As expected, levels of CdrA were elevated in a *wspF* background compared with PAO1 only (Fig. 3).

N-terminal sequencing was performed to gain insight into how CdrA is processed. The N-terminal sequence of the processed 150 kDa protein begins at amino acid 438. Were processing only to occur at the N-terminus, this would produce a 175 kDa protein, indicating that processing occurs at the C-terminus as well.

### CdrAB expression increases biofilm formation in wild-type and RSCV strains

**Fig. 4:**
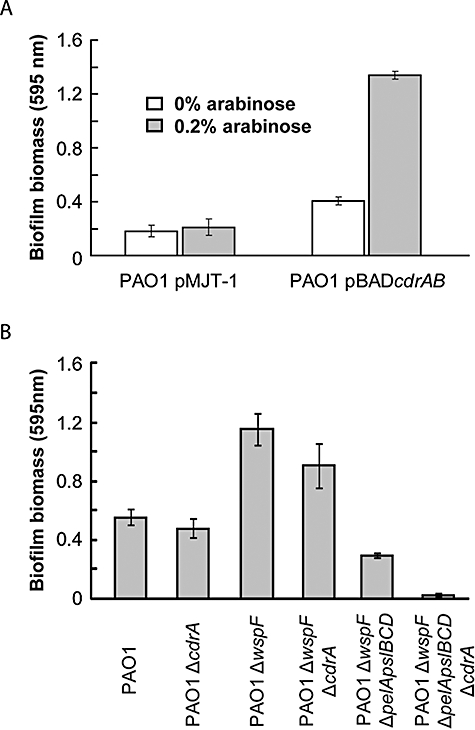
The effect of *cdrAB* expression on biofilm formation in a static microtitre dish assay. A. Biofilm formation as measured by crystal violet staining by a *cdrAB* overexpression strain. Open bars indicate control treatments without arabinose induction and grey bars indicate 0.2% arabinose-induced treatment. B. *cdrA* contributes to biofilm formation. Biofilm formation by *wspF, wspFcdrA*, *wspFpelApslBCD* and *wspFpelApslBCDcdrA* mutant strains were quantified by measuring crystal violet staining after 20 h of static growth in a microtitre dish assay.

The finding that CdrAB is co-regulated with other key matrix components, Pel and Psl, led us to hypothesize that it may contribute to the biofilm matrix. Therefore, we examined the effects of CdrAB on biofilm development. Initially, we evaluated the effects of *cdrAB* overexpression on biofilm formation. We constructed a plasmid, pBAD*cdrAB,* for arabinose-inducible expression of *cdrAB*. Biofilm formation was increased 6.4-fold when *cdrAB* was overexpressed as compared with the vector control (pMJT-1) (Fig. 4A).

To evaluate the impact of loss of CdrA expression on biofilm formation, we constructed in-frame deletions of *cdrA* in wild-type PAO1 and *wspF* backgrounds. This mutation had no effect on the RSCV colony morphology on solid medium (data not shown). In static microtitre-dish assays, a *ΔcdrA* mutation had minimal effect on accumulation of biofilm biomass in a PAO1 and a *wspF* background (Fig. 4B).

Exopolysaccharides have been shown to mask the effects of potential surface adhesins (Schembri *et al.*, 2004), so to eliminate potential interference from either the Pel or Psl polysaccharides we tested the effects of a *cdrA* mutation in a strain lacking Pel and Psl. Even though strains lacking Pel and Psl were substantially reduced in their ability to form biofilms (because Psl is important for surface attachment), the *wspFpelApslBCD* mutant produced 18-fold more biofilm biomass as compared with a *wspFpelApslBCDcdrA* strain (Fig. 4B).

**Fig. 5:**
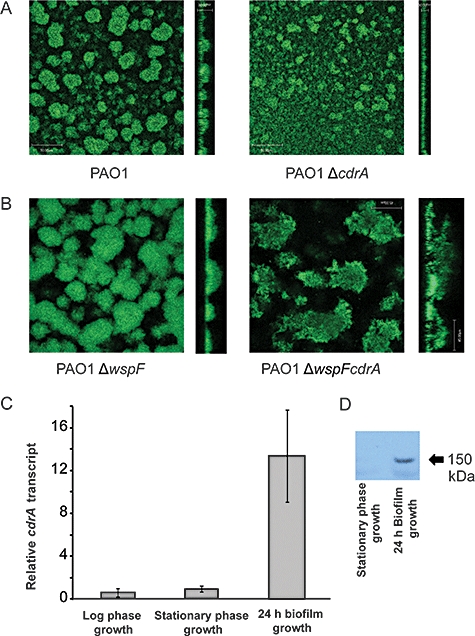
CdrA contributes to biofilm development under conditions of liquid flow. A. Top view and side view of PAO1 and PAO1*ΔcdrA* biofilms at 20× magnification. B. Top view and side view of PAO1 Δ*wspF* and PAO1 Δ*wspFcdrA* biofilms at 40× magnification. C. Relative transcript levels of *cdrA* for PAO1 cells grown planktonically for 24 h as compared with cells grown in a biofilm. D. Western blot analysis using CdrA antisera of PAO1 grown planktonically and as a biofilm for 24 h.

Next, we examined biofilm formation under conditions of continuous flow. Analyses of the *cdrAB* overexpression strain supported the microtitre-dish binding assay data. PAO1 pMJT-1 formed typical mushroom-shaped structures that are commonly observed for these conditions. In contrast, the *cdrAB* overexpression strain formed larger structures that were thicker and encompassed a greater volume of biofilm biomass (data not shown). However, unlike the *wspF* background which produced large compact cell aggregates (Fig. 5B), the biofilm formed by the *cdrAB* overexpression strain was characterized by more, but less tightly packed small cell aggregates.

The PAO1*cdrA* strain produced biofilms that were thinner and less structured than the wild-type (Fig. 5A). A *wspF* mutant produced biofilms typical of RSCV strains, characterized by a large amount of biomass and several large cell aggregates. A *cdrA* mutation in the *wspF* background had a dramatic phenotype. The biofilm cells were loosely packed, lacking the compact cell clusters of the *wspF* strain (Fig. 5B). Cells in the *wspF* strain were tightly associated with the biofilm, while in the *wspFcdrA* mutant strain, they were loosely associated and easily sloughed off. An illustration of this property is that *wspFcdrA* biofilms can be easily perturbed by changes in physical parameters such as flow. Simply interrupting flow through the flow cell reactor by periodically pinching the inlet tubing displaced the *wspFcdrA* biofilm (Movie S2), while the *wspF* biofilm (Movie S1) was not affected.

The flow cell data suggest that CdrA may play an important role in biofilms formed by both wild-type and *wspF* strains under conditions of flow. Previous expression analyses (Fig. 2) indicated that *cdrA* transcript levels were very low in wild-type planktonic culture. To determine if *cdrA* expression is elevated when PAO1 is grown as a biofilm, transcript levels were measured in cells grown under continuous flow in a tube biofilm reactor and compared with logarithmic and stationary phase planktonic cells. Transcript expression levels of *cdrA* in a biofilm were 15.4-fold higher than planktonic cells (Fig. 5C). These data indicate that *cdrA* is expressed during biofilm growth. Elevated CdrA expression in biofilm cultures of PAO1 was also observed in CdrA Western blots (Fig. 5D).

### CdrAB expression promotes auto-aggregation in liquid culture and small, congo-red binding colonies on solid medium

**Fig. 6:**
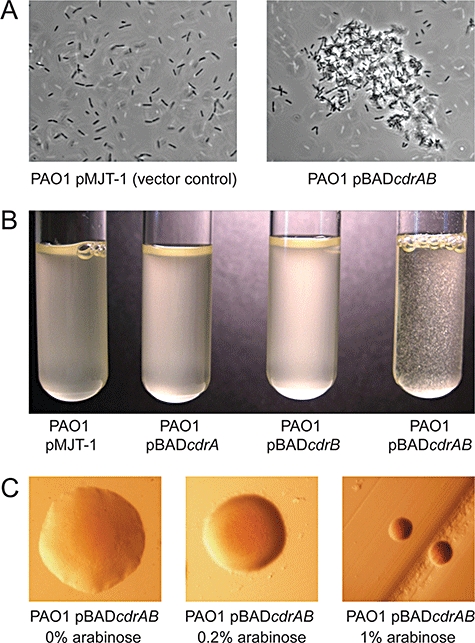
Overexpression of *cdrAB* results in aggregation in liquid culture and small colonies on solid medium. A. Phase contrast micrographs of *P. aeruginosa* PAO1 harbouring the expression vector pBAD*cdrAB* and pMJT-1. Bacteria were cultured in LB medium and 0.5% arabinose. B. Liquid cultures of *P. aeruginosa* PAO1 harbouring the expression vectors pBAD*cdrA*, pBAD*cdrB*, pBAD*cdrAB*, and pMJT-1 cultured in LB medium and 1.0% arabinose. C. Colonies of PAO1 harbouring the expression vector pBAD*cdrAB* cultivated on VBMM congo red agar plates supplemented with increasing concentrations of arabinose.

To gain further insight into CdrA function, we examined the effects of overexpression on *P. aeruginosa* phenotypes. Induction of *cdrAB* expression resulted in liquid culture auto-aggregation visible at the microscopic (Fig. 6A) and macroscopic level (Fig. 6B). Overexpression of *cdrA* or *cdrB* independently does not result in auto-aggregation. This is consistent with the data presented in Fig. 3, suggesting that CdrB is required for CdrA secretion.

Induction of *cdrAB* on solid medium results in a small colony phenotype that binds congo red. Increasing concentrations of arabinose result in a corresponding decrease in the size of the colony resulting in a small colony phenotype that is similar to an RSCV in size, but does not appear to be rugose (Fig. 6C). Overexpression was found to have no effect on growth, ruling out the possibility that *cdrAB* expression exerts a toxic effect on the cell (see Fig. S4). Another interpretation of a small colony phenotype is that CdrAB overexpression may reduce surface motility. Consistent with this observation, we demonstrated that CdrAB overexpression reduced *P. aeruginosa* swarming motility (see Fig. S5).

### CdrAB-mediated auto-aggregation requires the Psl polysaccharide

Although our data above indicate an important role for CdrA in biofilms, we had no mechanistic understanding. The liquid culture auto-aggregation associated with *cdrAB* overexpression provided our first clue. The presence of an N-terminal carbohydrate binding domain, and the co-regulation of *cdrAB* with the *pel* and *psl* gene clusters led us to propose that CdrA may bind to one of these polysaccharides. There is precedence for sugar binding in this particular family of adhesins (Menozzi *et al.*, 1994b). Like CdrA, expression of FHA of *B. pertussis* induces liquid culture auto-aggregation. FHA auto-aggregation can be disrupted by the exogenous addition of sugars to which it binds (Menozzi *et al.*, 1994a).

**Fig. 7:**
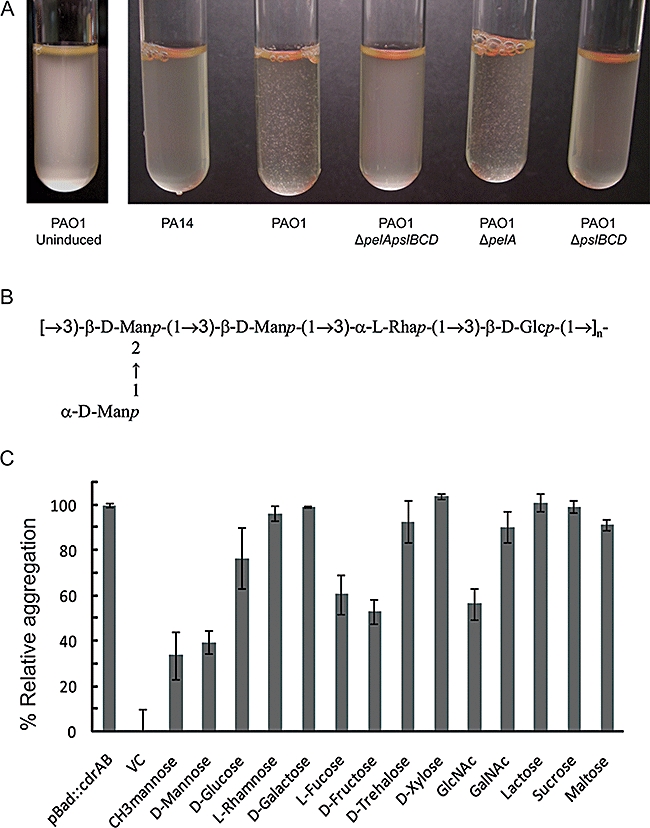
CdrA-mediated aggregation is dependent on Psl and inhibited by the addition of mannose. A. Aggregation of wild-type PAO1 and mutant strains that no longer produce the Pel and/or Psl exopolysaccharides were evaluated after induction of *cdrAB* with 1% arabinose and 3 h of growth. B. Structure of the Psl polysaccharide. C. Inhibition of aggregation by the addition of sugars. Analysis of the sugar-binding specificity of *cdrA* was monitored indirectly by measuring the % relative aggregation of PAO1 pBAD*cdrAB* strain in which *cdrAB* expression has been induced by addition of 1% arabinose.

We used the *cdrA*-mediated auto-aggregation phenotype (Fig. 6B) as a means to identify potential binding targets. As seen with FHA, we predicted that CdrA-binding targets would disrupt CdrA-dependent auto-aggregation. To test whether Pel and/or Psl could be binding targets, we examined the effect of *cdrAB* overexpression in *pel* and *psl* single and double mutant strains. Strains deficient in the production of Psl did not aggregate when *cdrAB* was overexpressed, indicating that CdrA may interact with the Psl polysaccharide under these conditions (Fig. 7A). PAO1 harbouring the vector control and strains containing the uninduced *cdrAB* overexpression construct did not auto-aggregate (data not shown).

One interpretation of these data is that a strain unable to make Psl is also impaired in production of CdrA. Using Western analysis, we demonstrated that CdrA levels in the supernatant were not affected by the *pslBCD* mutation (Fig. 3). However, cell-associated CdrA levels appeared to be lower. Conversely, using antisera specific to Psl, we also showed that a *cdrA* mutation did not significantly affect Psl production (see Fig. S6). We also evaluated aggregation in *P. aeruginosa* strain PA14, which lacks some of the genes required for Psl production (Friedman and Kolter, 2004a). Overexpression of *cdrAB* in this strain also failed to induce auto-aggregation (Fig. 7A).

### Mannose inhibits CdrA-mediated auto-aggregation

Previous studies have determined that Psl is a mannose-rich exopolysaccharide (Friedman and Kolter, 2004b; Ma *et al.*, 2007). Because Psl is a repeating pentasaccharide composed of mannose, rhamnose and glucose monosaccharides (in a 3:1:1 ratio), we surveyed these sugars and other selected sugars for the ability to disrupt CdrA-mediated auto-aggregation (Fig. 7B and C). Addition of D-mannose and methyl α-D-mannopyranoside reduced the relative auto-aggregation 61% and 66% respectively, as observed visually and quantified by measuring absorbance (Fig. 7C and Fig. S7). Inhibition of aggregation by mannose provides additional support for an interaction with the Psl exopolysaccharide. The other sugars found in Psl, L-rhamnose and D-glucose, had little effect on auto-aggregation.

Auto-aggregation was also diminished in the presence of other sugars. The addition of D- and L-Fucose, D-Fructose and GlcNAc resulted in a 40–46% decrease in relative aggregation. These results indicate that CdrA may be a multivalent adhesin that has the ability to recognize multiple carbohydrates. This is the case for FHA as well, which is capable of binding multiple, structurally distinct sugars. In every case tested, addition of sugars to *P. aeruginosa* not overexpressing *cdrAB* did not impact the aggregation state or growth of the culture (data not shown).

### Extracellular CdrA directly binds to Psl

**Fig. 8:**
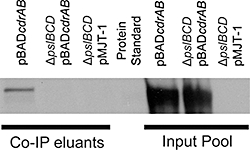
Psl and CdrA Co-immunoprecipitation analysis. This figure depicts a western analysis using CdrA antisera. The Co-IP eluants show what eluted off of Psl antisera coated beads after incubation with supernatants of selected strains. The input pool shows the amount of CdrA present in the supernatants that were incubated with the beads.

Data presented in Figs 6 and 7 indirectly suggest CdrA is capable of binding Psl. To directly test this hypothesis, we performed co-immunoprecipitation (Co-IP) experiments. We coated magnetic beads with anti-Psl antibodies. We then incubated these beads with cell free supernatant of *cdrAB* overexpressing strains in wild-type and *psl* mutant backgrounds. The rationale is that if CdrA is binding to extracellular Psl, when this Psl interacts with the Psl antibodies on the beads, CdrA will be co-precipitated. Protein eluted off the washed beads are then run on an SDS-PAGE and probed for CdrA using CdrA antisera. Figure 8 shows that CdrA failed to associate with the beads when *P. aeruginosa* was not producing Psl (PAO1 *pslBCD*/pBAD*cdrAB*), while it did in a strain producing Psl (PAO1/pBAD*cdrAB*). As a negative control, the same experiment was performed with a secreted, overexpressed protein, Hcp-1. This protein is a substrate of type VI secretion and is highly abundant in culture supernatant when overexpressed. This protein failed to associate with Psl in our assay (data not shown).

### CdrA is required for Psl localization in biofilms, contributing to their integrity

The Psl exopolysaccharide has been shown to be important for attachment to surfaces and maintenance of biofilm structure (Friedman and Kolter, 2004b; Jackson *et al.*, 2004; Matsukawa and Greenberg, 2004; Ma *et al.*, 2006). Because CdrA appears to bind Psl, we hypothesized that its function in a biofilm community might be to interact with Psl as part of the extracellular matrix, perhaps tethering cells to each other, Psl, or both.

**Fig. 9:**
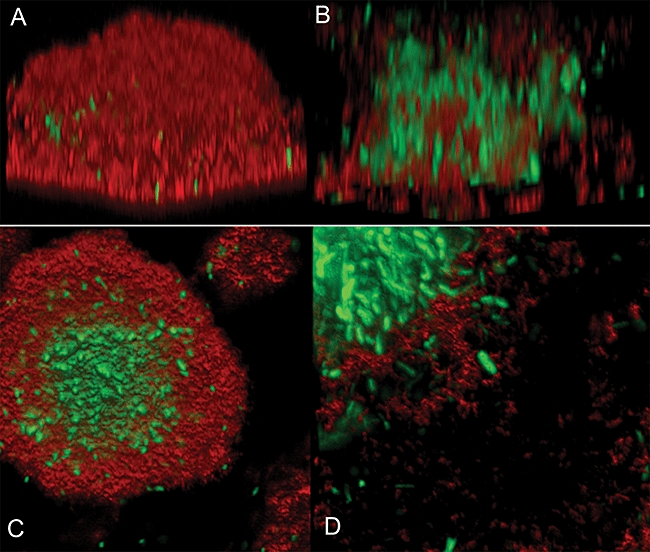
Lectin staining reveals CdrA is required for Psl association with a developing biofilm. Biofilms produced by *Pseudomonas aeruginosa* PAO1 Δ*wspF* (A, side view; C, bottom-up view) and Δ*wspFcdrA* (B, side view; D, bottom-up view) were stained with lectin. Bacterial cells were stained with Syto9 (green) and Psl stained with HHA-Tritc labelled lectin (red).

To test this hypothesis we examined the effects of a *cdrA* mutation on Psl distribution in a biofilm. Lectin staining techniques have been developed for the purposes of visualizing Psl. These lectins show specificity for the Manα1,3Man or Manα1,6Man structural components of the Psl EPS (Ma *et al.*, 2007). Consistent with previous results, a *wspF* strain produces a biofilm characterized by large cell aggregates in which the Psl forms a tightly associated shell around the aggregate (Fig. 9A and C) (Ma *et al.*, 2009). In sharp contrast, a *cdrA* mutation in this background produces a loosely structured biofilm in which the Psl is not tightly associated with the cell aggregates, and appears to be released into the flow stream (Fig. 9B and D). These results suggest a role for CdrA in the association and stabilization of Psl with bacterial cells and the biofilm matrix.

### Clinical isolates have the *cdrAB* genes and the capacity to functionally express CdrA

We also asked whether *P. aeruginosa* clinical isolates have the genetic capacity to produce CdrA. We isolated chromosomal DNA from 11 independent CF clinical isolates and assayed for the presence of the *cdrA* gene using PCR (Fig. S8). We found that all 11 isolates produced a band of the expected size. Wolfgang *et al.* also reported the presence of *cdrAB* in all 16 of their clinical and environmental isolates (Wolfgang *et al.*, 2003). We next tested whether cultures from the isolates were producing CdrA by western analysis. In this case six of the isolates produced a protein of about the right size that was immunoreactive with the CdrA specific antisera (Fig. S8). These data collectively suggest that *cdrA* is not specific to the PAO1 strain and could play a role in biofilm formation for some clinical strains.

## Discussion

In this study we have shown that *cdrAB* likely encodes a TPS system that is regulated by the second messenger c-di-GMP. In a *wspF* mutant strain, characterized by high cellular levels of c-di-GMP, *cdrA* expression is elevated. Conversely, depletion of c-di-GMP results in a corresponding decrease in *cdrA* expression. Modelling experiments and bioinformatics predict that CdrA is a rod-shaped protein with a β-helical tertiary structure and an exposed RGD integrin binding motif. CdrA also contributes to biofilm formation under conditions of flow. In the *wspF* background, biofilm produced by a *cdrA* mutant strain is characterized by a loss of biofilm integrity. The overexpression of *cdrAB* results in the formation of Psl-dependent liquid culture aggregates, suggesting that CdrA may bind Psl. Additional evidence supporting CdrA binding Psl is the inhibition of aggregation by addition of mannose, a monosaccharide component of Psl. Lectin staining for Psl distribution in the biofilm revealed that a mutation in *cdrA* affected the distribution of Psl, producing a matrix that was loosely associated with biofilm cells and a biofilm of reduced structural integrity. Our data suggest that CdrA is an integral protein component of the biofilm matrix that provides structural stability by binding Psl.

We found that *cdrA* expression was directly responsive to changing cellular c-di-GMP levels (Fig. 2). Artificial elevation or depletion of c-di-GMP levels through the expression of the cyclase encoded by PA1120 or the phosphodiesterase encoded by PA2133, raised and lowered *cdrA* expression respectively. This is consistent with the finding that *cdrA* expression is controlled by the transcriptional regulator FleQ. In this model, FleQ binds to the *cdrAB* promoter, repressing transcription. When c-di-GMP levels rise, it binds to FleQ causing it to fall off its DNA binding site, relieving transcriptional repression. One interesting result pertains to the levels of CdrA expression in a *wspF* strain, compared with the *wspFpelApslBCD* triple mutant. The expression levels of *cdrA* are much lower in the later. Why this occurs is not clear. Perhaps a careful quantification of c-di-GMP levels in the strain will reveal that they are lower in the triple mutant, resulting in less dissociation of the repressor FleQ from *cdrAB* promoter DNA. We are currently investigating this possibility. We also found that *cdrA* transcription was induced in PAO1 when grown as a biofilm (Fig. 5C). Although the mechanism underlying this result is not clear, it demonstrates one condition where expression is induced in a wild-type strain. In addition, this supports the argument that CdrA function relates to structural integrity of the biofilm EPS matrix.

In several Gram-negative species of bacteria, the intracellular signal c-di-GMP promotes the biofilm lifestyle, by downregulating motility and upregulating the production of secreted polysaccharides. A few examples include the production of the vps exopolysaccharide by *Vibrio cholerae*, and cellulose production in *E. coli*. *P. aeruginosa* also uses c-di-GMP to positively regulate production of the key biofilm polysaccharides, Pel and Psl. A novel finding from our study is that c-di-GMP also promotes biofilm formation by inducing expression of matrix functions other than exopolysaccharides. This is also the first report of a protein structural component of the biofilm matrix for *P. aeruginosa*. It will be interesting to see if similar adhesins whose expression are controlled by c-di-GMP also function as matrix stabilizers in other species.

CdrA, like many adhesins of the TPS family, appears to be exported and processed. The gene is predicted to encode a 220 kDa protein. Initial Western analysis indicated that CdrA is post-translationally processed. An approximately 150 kDa immunoreactive band is observed in the supernatant of strains expressing CdrA (Fig. 3). There are other bands below the 150 kDa product, including a faint band directly below the 150 kDa band, but they appear to be proteolytic break down products. This is common for this class of proteins. Initial analysis of this processed 150 kDa form of CdrA suggests that it is cleaved at both the N- and C-terminus. N-terminal sequencing indicates that 45 kDa is cleaved off of the N-terminus. This would lie somewhere close to the carbohydrate binding domain. This could be a key step in activating the Psl-binding function of CdrA. The significance of the cell-associated CdrA is not clear. One possibility is that it represents aggregates which occur when CdrA is overexpressed and the aggregates spin down with the cells after centrifugation.

The relationship between CdrA and Psl in the biofilm matrix is a key consideration. The evidence of both extracellular and cell-associated CdrA and Psl calls into question what the functional arrangement is within the biofilm matrix. There are a number of possibilities that are not mutually exclusive. Secreted CdrA could act as a cross-linker of individual strands of Psl within the matrix. This would reinforce the matrix polymers and provide integrity to the matrix (which is apparent in the supplementary movie files). Alternatively, cell-associated CdrA may serve to anchor the cells to Psl in the matrix. The data presented in Fig. 9 might support both possibilities, as Psl-stainable material is seen to be less densely packed and not as tightly cell-associated. If the major function of CdrA is to promote biofilm integrity, it is easy to imagine why the *cdrA* mutant strains had a pronounced impact on biofilm structure under flow conditions and not static conditions.

Most β-helix adhesins of the FHA family are thought to play a role in bacterium-eukaryotic cell interactions in the context of pathogenesis. Indeed, the integrin binding domain of CdrA suggests that this adhesin might also mediate interactions with the eukaryotic extracellular (ECM) matrix. Upregulation of CdrA by RSCVs and wild-type biofilms in the CF environment may have some interesting consequences. Besides stabilizing the biofilm, CdrA may promote interactions with host cells. The finding that additional sugars also interfere with CdrA-mediated aggregation (Figure 7C) indicates that it could be a multivalent adhesin with multiple binding targets. However, the fact that CdrA is upregulated in biofilm cultures in the absence of a host (both in wild-type and RSCV strains) suggests that it functions in biofilm matrix stabilization. Interestingly, the genome of *P. aeruginosa* PAO1 contains five large ORFs besides *cdrA* that encode proteins with similarity to FHA from *B. pertussis*, which have yet to be functionally characterized (Croft *et al.*, 2000).

This study also gives one pause to consider the organization and structure of the EPS matrix. Traditional thinking suggests the matrix is a random distribution of secreted polymers in which the cells are passively imbedded. This point of view is beginning to change. Previous studies of Psl have shown that it forms a discrete shell around the periphery of biofilm cell aggregates, presumably holding the interior cells in (Ma *et al.*, 2007, 2009). Our findings indicate that CdrA is another structural component of that shell. Thus, emerging evidence indicates the biofilm matrix is much more complex and ordered than previously suspected.

Such complexity is observed in eukaryotic organisms, where the ECM provides the structural support necessary for multicellular organization and co-ordinated function. This is accomplished in large part by polysaccharides and fibrous proteins. The ECM matrix provides several functions besides just structure, for example it can signal the resident cells residing within. It is possible that signalling mediated by c-di-GMP and the temporal and spatial control of matrix biosynthesis are analogous to the signalling and co-ordinated organization that functions in the biosynthesis of ECM by multicellular organisms. Studies of the Wsp signal transduction system suggest that the production of c-di-GMP is stimulated in response to growth on a surface (Guvener and Harwood, 2007). The activation of WspR results in the catalysis of c-di-GMP, increased cellular levels of c-di-GMP and increased biofilm formation because of increased expression of Pel and Psl polysaccharides (Hickman *et al.*, 2005). CdrA may function in anchoring the matrix via its interaction with the Psl polysaccharide to provide structural support between cells within a biofilm analogous to the function of fibrous proteins in the ECM of eukaryotes. Similarly, it remains to be seen whether individual matrix components may be capable of transducing signals to the cells within.

Future work will determine if CdrA and other TPS systems interact with additional matrix components derived by *P. aeruginosa* or matrix components of other organisms in the various environmental niches they occupy including hosts. Studies in our lab are directed at understanding the interaction of CdrA with epithelial cells and characterizing the functional role of the RGD integrin binding motif. Random and site-directed mutagenesis is currently underway to identify the key structural and functional protein residues and carbohydrate recognition domains. Proteins that belong to the TPS family have been shown to be multivalent adhesins. Our results also suggest that multiple carbohydrates may interact with CdrA and a mutational analysis of *cdrA* combined with a carbohydrate binding array will be used to further delineate carbohydrates that interact with CdrA.

## Experimental procedures

### Bacterial strains and growth conditions

Strains and plasmids used for this study are listed in Table 1. Bacteria were grown overnight at 37°C with shaking at 250 r.p.m. in Luria–Bertani (LB) broth unless otherwise stated. L-arabinose at the reported concentrations or 0.5 mM IPTG was supplemented into the medium for inducible-expression experiments. Motility plate assays were used to determine differences in swarming motility. LB agar medium was solidified with 0.45% Noble agar. Plates were inoculated using a sterilized platinum wire with log-phase cells and swarming diameter was measured 40 h after incubation at 30°C.

**Table 1:** List of strains.

Strain or Plasmid	Relevant characteristics	Source or reference
*Pseudomonas aeruginosa PA14*	Wild-type	Rahme *et al.*, 1995
*P. aeruginosa PAO1*	Wild-type	Holloway, 1955
*ΔwspF*	*ΔwspF*; markerless	Hickman *et al.*, 2005
*ΔpelA*	*ΔpelA*; markerless	Starkey *et al.*, 2009
*ΔpslBCD*	*ΔpslBCD*; markerless	Kirisits *et al.*, 2005
*ΔpelApslBCD*	*ΔpelApslBCD*; markerless	This study
*ΔwspFcdrA*	*ΔwspF ΔcdrA*; markerless	This study
*ΔwspFpelApslBCD*	*ΔwspF ΔpelA ΔpslBCD*; markerless	This study
*ΔwspFpelApslBCDcdrA*	*ΔwspF ΔpelA ΔpslBCD ΔcdrA*; markerless	This study
*ΔpppA hcp1-V*	Hcp1 constitutive expression, VSVg tagged Hcp1	Hsu *et al.*, 2009
CF 23	Cystic Fibrosis clinical isolate	Jane Burns
CF 47	Cystic Fibrosis clinical isolate	Jane Burns
CF 71	Cystic Fibrosis clinical isolate	Jane Burns
CF 102	Cystic Fibrosis clinical isolate	Jane Burns
CF 114	Cystic Fibrosis clinical isolate	Jane Burns
CF 116	Cystic Fibrosis clinical isolate	Jane Burns
CF 127	Cystic Fibrosis clinical isolate	Jane Burns
CF 153	Cystic Fibrosis clinical isolate	Jane Burns
CF 166	Cystic Fibrosis clinical isolate	Jane Burns
CF 184	Cystic Fibrosis clinical isolate	Jane Burns
Plasmids		
pMRP9-1	pGFPmut2 pUCP18	Davies *et al.*, 1998
pMJT-1	araC-PBAD cassette of pJN105 (Newman & Fuqua, 1999) cloned in pUCP18, Amp^R^ (Carb^R^)	Kaneko *et al.*, 2007
pET-20b(+)	Protein expression vector, Amp^R^	Novagen
pJN105	*araC-PBAD* cassette cloned in pBBR1MCS-5; Gm^r^	Newman & Fuqua, 1999
pJN2133	PA2133 cloned into pJN105; Gm^r^	Hickman *et al.*, 2005
pJN1120	PA1120 cloned into pJN105; Gm^r^	Hickman and Harwood, 2008
pEX18gm	Allelic exchange vector	Hoang *et al.*, 1998
pDelWspF	*wspF* deletion construct	Hickman *et al.*, 2005
pMPELA	Pel deletion construct	Starkey *et al.*, 2009
pEX4625	*cdrA* deletion construct	This study
pBADcdrAB	*cdrAB* cloned into pMJT-1	This study
pBADcdrA	*cdrA* cloned into pMJT-1	This study
pBADcdrB	*cdrB* cloned into pMJT-1	This study
pBB022	*cdrA* fragment cloned into pET-20b(+)	This study

### Strain and plasmid construction

DNA manipulations were performed using standard methods. Genomic DNA was prepared using the DNeasy Blood & Tissue Kit (QIAGEN). PCR amplicons were excised and purified from agarose gels by gel extraction with the Qiaex II kit (QIAGEN). Deletion mutants of *pelA, pslBCD, wspF* and *cdrA* were constructed by allelic exchange using standard procedures for *P. aeruginosa*. Plasmids engineered with the deletion constructs were mated into *P. aeruginosa* and selected on VBMM agar (Vogel and Bonner, 1956) containing 100 µg ml^−1^ gentamicin. Strains were further selected on LB agar plates containing 5% sucrose (w/v) to isolate double recombinants. Deletion mutants were confirmed by PCR with primer sets internal and external to the deletion. Arabinose inducible *cdrA*, *cdrB* and *cdrAB* plasmids were constructed by PCR amplification and cloned into the multicloning site of the arabinose-inducible expression vector, pMJT-1. XbaI and EcoRI restriction sites were engineered into the PCR primers to directionally clone each amplicon.

### Isolation of total RNA, RT-PCR and evidence that *cdrAB* is an operon

Cells were grown in LB broth at 37°C overnight for RT-PCR. Tube biofilm experiments were conducted as previously described (Schaefer *et al.*, 2001; Kirisits *et al.*, 2005). *P. aeruginosa* PAO1 was injected into 6.35 mm inner-diameter tube and incubated for 30 min. Control cells were incubated staticly. Biofilms were grown at room temperature in LB medium containing 100 mM MOPS at 50 ml h^−1^ flow rate for 48 h.

Total RNA was isolated by using the RNAprotect Bacteria Reagent and RNeasy kit (QIAGEN). Genomic DNA was eliminated by RQ1 RNase-Free DNase (Promega) treatment during the isolation procedure. Reverse transcription was performed on 1 µg of RNA by using the SuperScriptIII First-Strand Synthesis System (Invitrogen) according to the manufacturer's recommendations. Real-Time PCR was performed with the 7500 Real-Time PCR System with the SYBR Green PCR Master Mix (Applied Biosystems). Cycling parameters were 95°C for 10 min, followed by 40 cycles of 95°C for 15 s and 60°C for 1 min. Standard curves for quantification of transcripts were performed using dilutions of *P. aeruginosa* chromosomal DNA from 1 X 10^−4^ ng to 10 ng (Lequette *et al.*, 2006). Transcript levels of all genes tested were normalized to transcript levels of the *ampR* gene (Lequette *et al.*, 2006). PCR reactions were performed in quadruplicate for each gene. Data represent the average and the standard deviation between samples.

Transcription of *cdrAB* as an operon was verified by RNA extraction and cDNA synthesis as described above. PCR from cDNA was utilized to amplify an 854 bp region spanning the N-terminus of *cdrA* and the C-terminus of *cdrB.* PCR experiments included reactions consisting of cDNA, genomic DNA, RNA and cDNA no reverse transcriptase controls.

### Modelling experiments

CdrA is a 2154 amino acid protein. *De novo* modelling programmes or servers are not reliable at such sizes. To overcome this limitation, CdrA was modelled as six separate segments and reattached by structural alignments of overlapping domains. Three different sets of segment structures were used to assemble CdrA structures. Three whole protein models were made from each set of segment models, each with a different set of overlapping regions. Thus, nine total models were made with this protocol. The top models out of these nine were chosen by a recent version of the residue-specific all-atom probability discriminatory function (RAPDF) (Samudrala and Moult, 1998).

Domain boundaries were predicted by the DomPred algorithm on the 3D Jury server (Marsden *et al.*, 2002). Segment structures were modelled using the I-TASSER server. The I-TASSER algorithm is an enhanced threading algorithm and is the most reliable *de novo* modelling technique as of the CASP8 competition (http://www.predictioncenter.org/casp8) (Wu *et al.*, 2007; Zhang, 2008). After each segment was attached, the resulting structure was decreased in potential energy by a combination of the side-chains with a Rotamer Library (SCWRL3) algorithm (Canutescu *et al.*, 2003) and the Energy Calculations and Dynamics Program (ENCAD) (Levitt *et al.*, 1995). A trinary confidence measure was implemented by comparing the results of sequence based secondary structure prediction to secondary structure elements assigned to the model. The sequence based secondary structure predictions were performed by PsiPred (Bryson *et al.*, 2005). The model based secondary structure assignments were performed by the Definition of Secondary Structure of Proteins (DSSP) algorithm (Kabsch and Sander, 1983). Amino acids that had matching secondary structure assignments were denoted with a high confidence. Amino acids in which a secondary structure prediction was made by one method but not the other was denoted with an intermediate confidence. Amino acids in which opposite secondary structures assigned by the two methods were denoted with a low confidence.

Three separate sets of segments were modelled using the I-TASSER server. For each set of segments, three different arrangements of overlapping regions were used to build the whole protein. Nine different models were produced by repeating this method with altered parameters. The best of these nine final models was chosen by an all-atom scoring function (Samudrala and Moult, 1998) The RAPDF (Samudrala and Moult, 1998) was used to rank these models by likelihood of accuracy. Our two highest scoring models had a similar overall tertiary structure. We prefer the second highest scoring model because of a structurally independent putative signal peptide and an exposed putative integrin binding domain. Confidence of the model was determined by comparing the secondary structure that was predicted from the model with the secondary structure inferred from the sequence (Fig. 1B) (Kabsch and Sander, 1983). Secondary structure predictions were identical for 57% of the model.

### Generation of CdrA antibody and Western blot detection

A unique domain of the *cdrA* gene (bp 2308–2808) from *P. aeruginosa* PAO1 was amplified by PCR and cloned into pET-20b(+) vector (Novagen) to construct plasmid pBB022 for the expression of His-tagged CdrA protein (aa 770–936). Protein expression was induced at 16°C by addition of 0.5 mM IPTG at 0.5 OD_600_ and subsequent overnight incubation. The recombinant CdrA protein was purified under denaturing conditions by affinity chromatography on Ni-NTA resin as recommended by the manufacturer (Qiagen). Rabbit polyclonal antibodies against CdrA (αCdrA) were raised in New Zealand white rabbits by immunization (Express Line Plus Protocol) of each rabbit with 2 mg of recombinant CdrA protein (Lampire). Polyclonal antibody against CdrA was obtained.

For immunoblotting experiments, overnight cultures of *P. aeruginosa* were inoculated into medium as indicated and grown to mid-log phase. Whole cell lysates and proteins concentrated from supernatants were prepared as described by Mougous *et al.* with modification (Mougous *et al.*, 2006). Bacterial cells were harvested by centrifugation, resuspended in 50 µl of sample buffer mixed with 100 µl of Laemmli buffer and heated to 99°C for 5 min. The protein concentration of each whole cell lysate sample was measured with the Pierce® 660nm protein assay (Thermo Scientific) and 10 µg of protein of the respective sample was used for electrophoresis, blotted and incubated with αCdrA (1:2000). Proteins residing in the supernatant were concentrated from 1.6 ml of supernatant by TCA precipitation (Mougous *et al.*, 2006). Normalization and sample loading were based on the corresponding protein concentration of whole cell lysates from which the supernatants were derived. Detection was performed with the SuperSignal West Pico Chemiluminescent Substrate (Thermo Scientific).

### Western immunoblotting of polysaccharide extracts from colony biofilms

Colony biofilms were grown on sterile 0.22 Micron polycarbonate filters (GE Water & Process Technologies) on LB agar medium for 48 h (Rani *et al.*, 2007). Filters were transferred to fresh medium daily. Bacterial samples from two filters were resuspended in 100 µl of 0.5 M EDTA and boiled for 5 min and processed. Supernatants were collected and the protein levels were quantified by Bradford Assay. Samples were additionally treated with proteinase K (final concentration 0.5 mg ml^−1)^ for 60 min at 60°C and 30 min at 80°C. Polysaccharide extracts were normalized based on protein concentration of samples prior to treatment with proteinase K and 10 µg of samples were spotted on a nitrocellulose membrane and allowed to air dry for 30 min. Immunoblots were probed with α-PSL (Byrd *et al.*, 2009).

### Co-IP of CdrA bound to Psl polysaccharide

Supernatants were buffered to a final concentration of 1× PBS and 1× Roche protease inhibitor EDTA-free cocktail. Protein was concentrated from buffered supernatants by centrifugation filtration (Amicon Ultracel-10K). Dynabeads Protein A magnetic beads (Invitrogen) were incubated with 5 µl of α-PSL (Byrd *et al.*, 2009) according to the manufacturer's recommendations with the following modifications. Beads were washed twice after antibody binding with PBS Tween 20 and the target antigen was incubated 20 min with the Dynabeads-antibody complex. Target antigen was eluted with 20 µl of 2× Laemmli buffer and separated by SDS PAGE. Western blot analysis was performed with α-CdrA. To verify that equal amounts of CdrA were present in the input pools of strains overexpressing CdrA, supernatants were TCA precipitated from the various treatments and included as an additional control. Additionally, the levels of the secreted protein Hcp1 were evaluated to determine the specificity of Co-IP with a non-target antigen.

### Static microtitre biofilm formation assays

Static biofilm formation was evaluated by monitoring biofilm biomass accumulation using the method of O'Toole and Kolter (1998a,b); with minor modifications. Overnight cultures were subcultured and grown to mid-log growth phase. Nunc Bacti 96 well microtitre plates containing 95 µl of VBMM medium were inoculated with and 5 µl of culture and incubated for 20 h at 37°C under static conditions. Plates were washed to remove nonadherent cells by submerging in deionized water and decanting. Biofilm biomass was stained by the addition of 150 µl of 0.1% (w/v) crystal violet added to each well, incubated for 15 min, rinsed with water, and solubilized by the addition of 200 µl of 95% ethanol. Absorbance was measured at 595 nm. Data represent the mean of six replicates.

### Biofilm flow cell experiments

Biofilms were cultivated at 37°C for 48 h in polycarbonate flow cells with individual channel dimensions of 1 by 4 by 40 mm in 1% LB (v/v) medium. Flow cells were inoculated with 100 µl of bacterial culture at an OD600 of 0.05 in 1% LB medium diluted from exponentially growing cultures. Bacterial cells were allowed to attach to the inverted glass coverslip for 1 h before the initiation of medium flow at a constant rate of ∼3.75 ml h^−1^ using a Watson Marlow 205S peristaltic pump (Watson Marlow, Falmouth, UK).

### Aggregation and sugar analysis

Stationary-phase cultures were diluted 30-fold into medium supplemented with 1% (w/v) arabinose, 300 µg ml^−1^ carbenicillin and various sugars at a final concentration of 5 mg ml^−1^. Sugars were obtained from commercial sources. Aggregation was evaluated by visual assessment and/or measurement of the absorbance at 600 nm after 3 h of growth. The % relative aggregation was calculated by dividing the treatment by the average of the control strain and multiplying by 100.

### Lectin staining

Biofilms were stained with TRITC-labelled HHA lectin (EY laboratories), and SYTO 9 (Invitrogen) and visualized at 63× magnification by CLSM after 48 h of growth at 37°C. The specificity of the HHA lectin for Psl has been previously described (Ma *et al.*, 2009). Flow was suspended and 0.3 ml of 0.02 mM SYTO9 and 100 µg ml^−1^ TRITC-HHA was injected upstream of the inlet flow. Biofilms were stained for 15 min in the dark and examined by confocal laser scanning microscopy (CLSM) 15 min after flow was resumed (Hentzer *et al.*, 2001).
