# Supplementary material for: Pseudomonas aeruginosa uses a cyclic-di-GMP-regulated adhesin to reinforce the biofilm extracellular matrix
Source: Mol Microbiol. 2010 Jan 17;75(4):827–42. doi: 10.1111/j.1365-2958.2009.06991.x (PMC2847200; doi:10.1111/j.1365-2958.2009.06991.x)
Supplement: Supplementary file 1 [file mmi0075-0827-SD1.pdf]

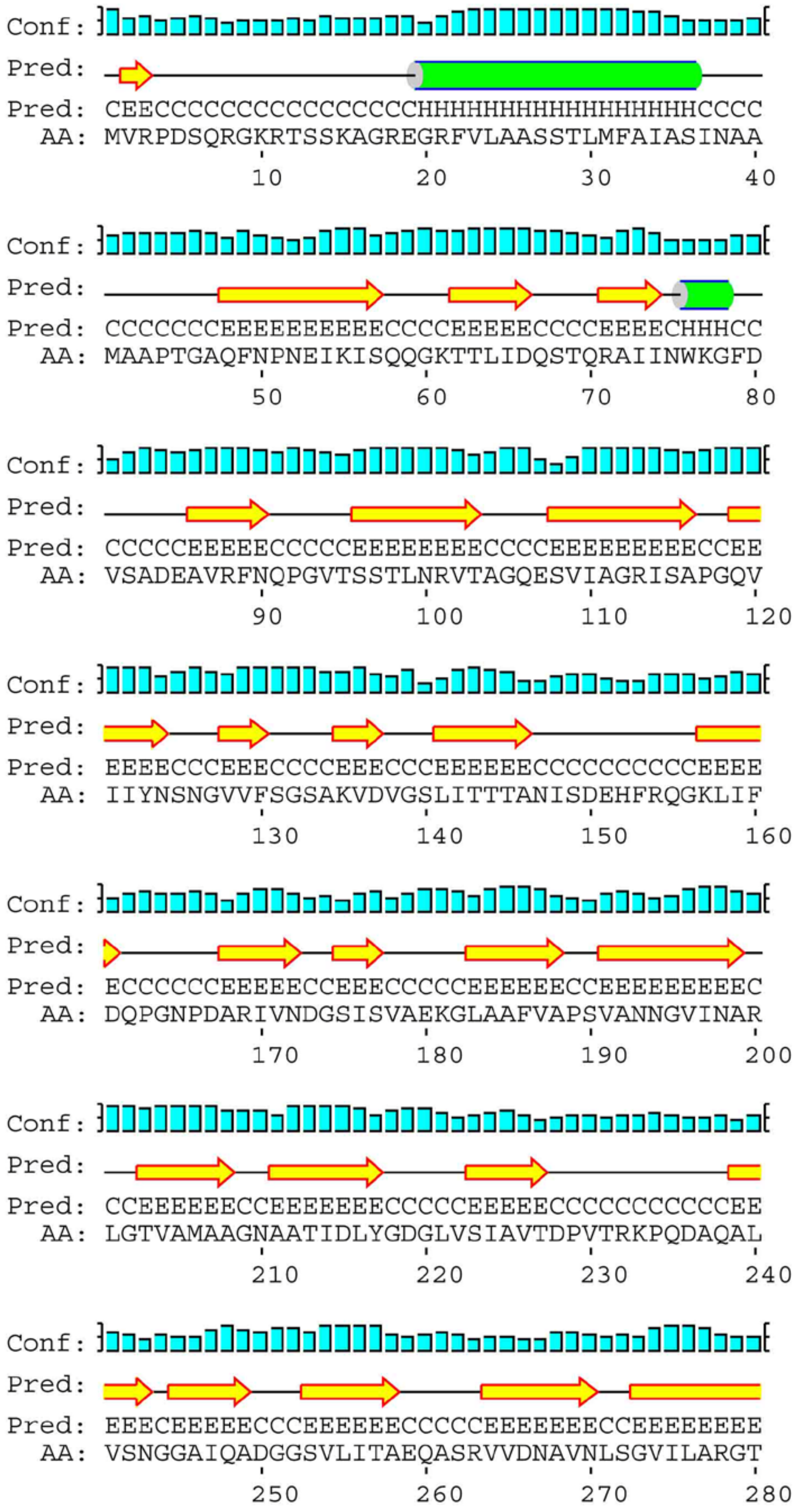

Conf:
Pred:
Pred: CEECCCCCCCCCCCCCCCCHHHHHHHHHHHHHHHHHCCCC
AA: MVRPDSQRGKRTSSKAGREGRFVLAASSTLMFAIASINAA
10 20 30 40
Conf:
Pred:
Pred: CCCCCCCEEEEEEEEEECCCCEEEEECCCCEEEECHHHCC
AA: MAAPTGAQFNPNEIKISQQGKTTLIDQSTQRAIINWKGFD
50 60 70 80
Conf:
Pred:
Pred: CCCCCEEEEECCCCCEEEEEEEECCCCEEEEEEEEECCEE
AA: VSADEAVRFNQPGVTSSTLNRVTAGQESVIAGRISAPGQV
90 100 110 120
Conf:
Pred:
Pred: EEEECCCEEECCCCEEECCCEEEEEECCCCCCCCCCEEEE
AA: IIYNSNGVVFSGSAKVDVGSLITTTANISDEHFRQGKLIF
130 140 150 160
Conf:
Pred:
Pred: ECCCCCCEEEEECCEEECCCCCEEEEEECCEEEEEEEEEC
AA: DQPGNPDARIVNDGSISVAEKGLAAFVAPSVANNGVINAR
170 180 190 200
Conf:
Pred:
Pred: CCEEEEEECCEEEEEEECCCCCEEEEECCCCCCCCCCCEE
AA: LGTVAMAAGNAATIDLYGDGLVSIAVTDPVTRKPQDAQAL
210 220 230 240
Conf:
Pred:
Pred: EEECEEEEECCCEEEEEECCCCCEEEEEEECCEEEEEEEE
AA: VSNGGAIQADGGSVLITAEQASRVVDNAVNLSGVILARGT
250 260 270 280

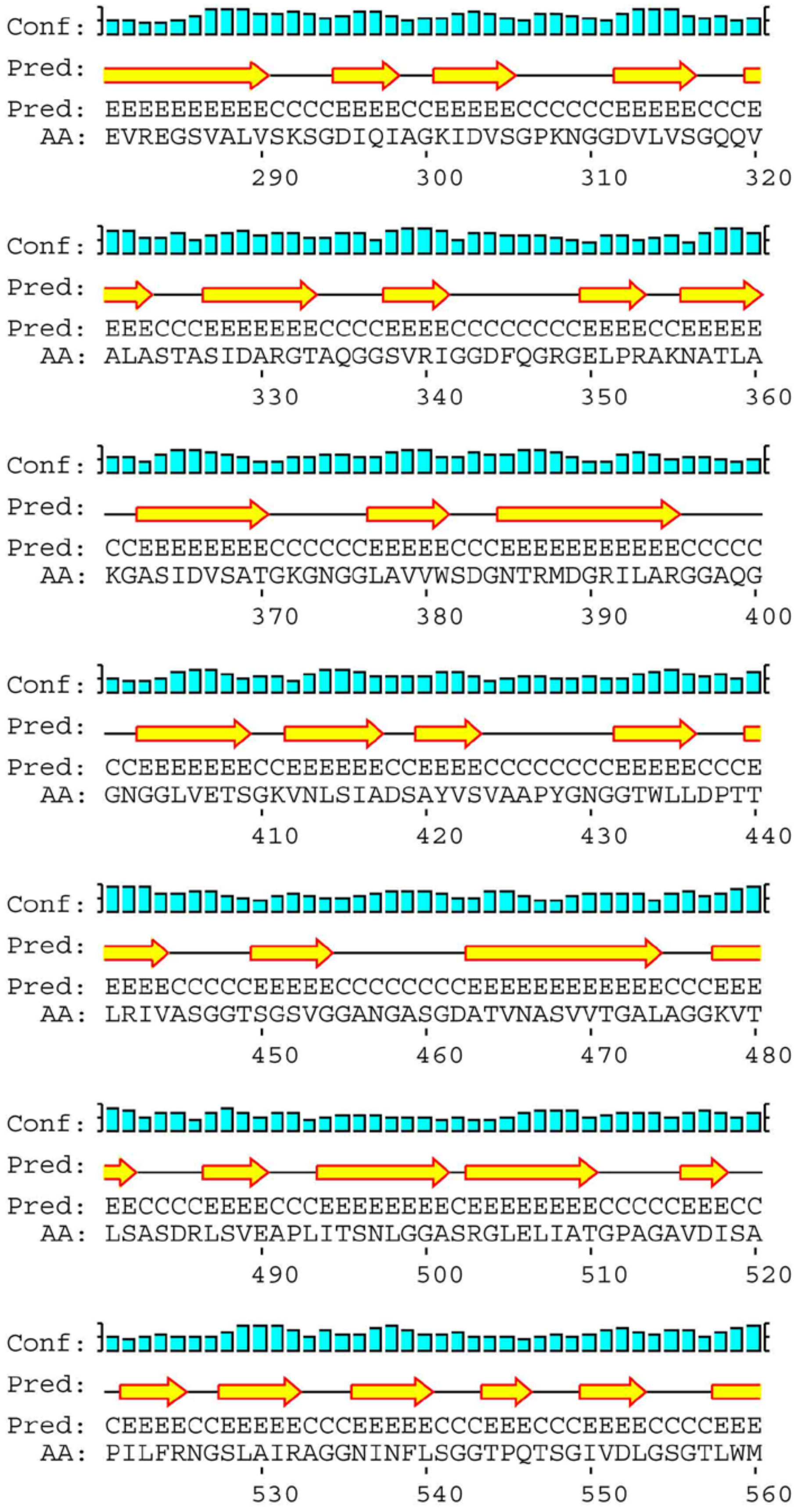

Conf:
Pred:
Pred: EEEEEEEEEECCCCEEEECCEEEEECCCCCCEEEEECCCE
AA: EVREGSVALVSKSGDIQIAGKIDVSGPKNGGDVLVSGQQV
290 300 310 320
Conf:
Pred:
Pred: EEECCCEEEEEEECCCCEEEECCCCCCCCEEEECCEEEEE
AA: ALASTASIDARGTAQGGSVRIGGDFQGRGELPRAKNATLA
330 340 350 360
Conf:
Pred:
Pred: CCEEEEEEEECCCCCCEEEEECCCEEEEEEEEEEEECCCCC
AA: KGASIDVSATGKGNGGLAVVWSDGNTRMDGRILARGGAQG
370 380 390 400
Conf:
Pred:
Pred: CCEEEEEEEECCEEEEEEECCEEEECCCCCCCCEEEEECCCE
AA: GNGGLVETSGKVNLSIADSAYVSVAAPYGNGGTWLLDPTT
410 420 430 440
Conf:
Pred:
Pred: EEEECCCCCEEEEECCCCCCCCEEEEEEEEEEEECCCEEE
AA: LRIVASGGTSGSVGGANGASGDATVNASVVTGALAGGKVT
450 460 470 480
Conf:
Pred:
Pred: EECCCCEEEECCCEEEEEEEEECEEEEEEEEECCCCCEEECC
AA: LSASDRLSVEAPLITSNLGGASRGLELIATGPAGAVDISA
490 500 510 520
Conf:
Pred:
Pred: CEEEECCEEEEECCCEEEEEECCCEEECCCEEEEECCCCEEE
AA: PILFRNGSLAIRAGGNINFLSGGTPQTSGIVDLGSGTLWM
530 540 550 560

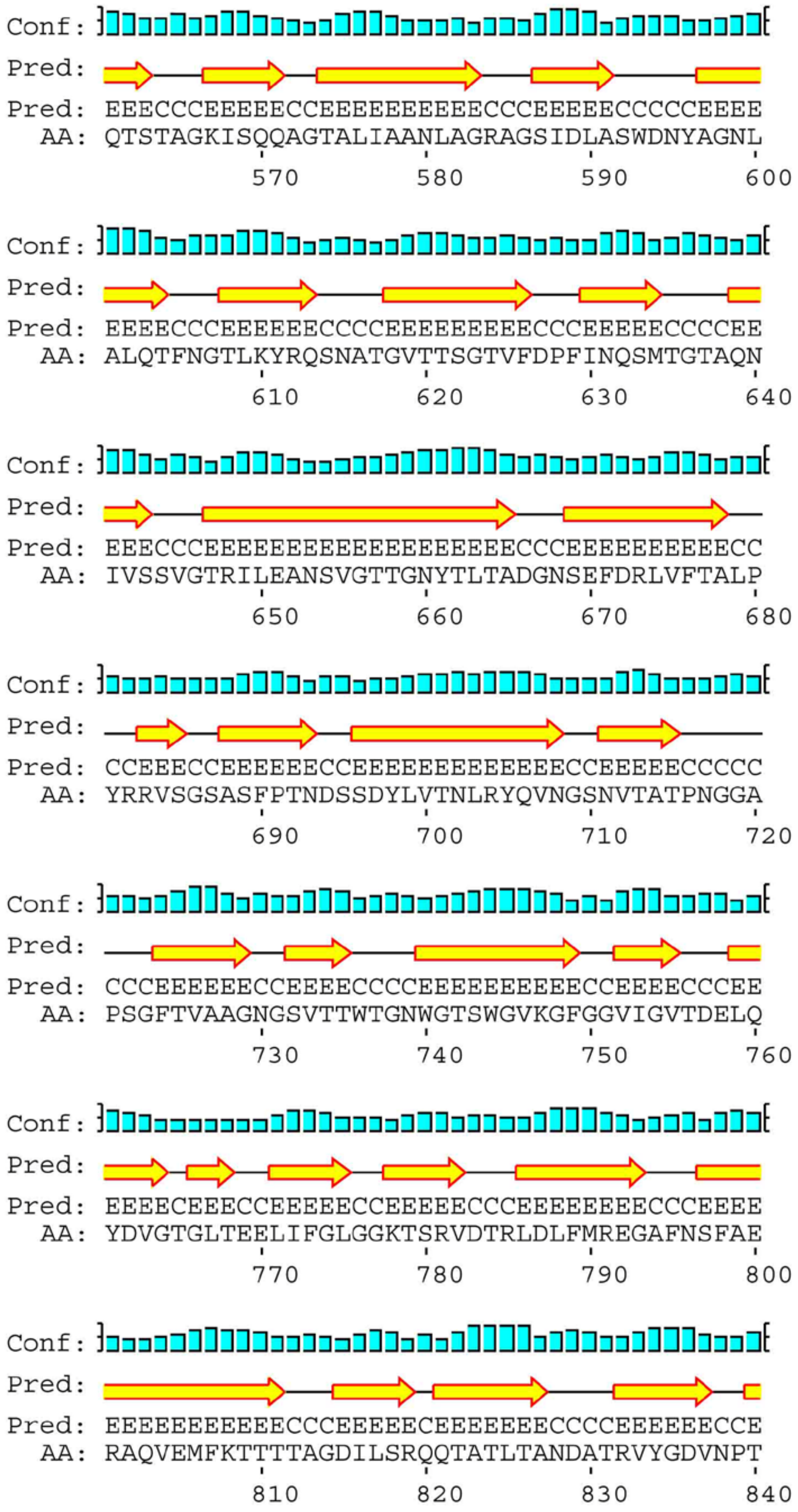
Conf:
Pred:
Pred: EEECCCEEEEECCEEEEEEEEEEECCEEEEEECCCCEEEE
AA: QTSTAGKISQQAGTALIAANLAGRAGSIDLASWDNYAGNL
570 580 590 600
Conf:
Pred:
Pred: EEEECCCEEEEEECCCCEEEEEEEEECCCEEEEECCCCEE
AA: ALQTFNGTLKYRQSNATGVTTSGTVFDPFINQSMTGTAQN
610 620 630 640
Conf:
Pred:
Pred: EEECCCEEEEEEEEEEEEEEEEEEECCCEEEEEEEEEECC
AA: IVSSVGTRILEANSVGTTGNYTLTADGNSEFDRLVFTALP
650 660 670 680
Conf:
Pred:
Pred: CCEEECCEEEEEECCEEEEEEEEEEEEECCEEEEECCCCC
AA: YRRVSGSASFPTNDSSDYLVTNLRYQVNGSNVTATPNGGA
690 700 710 720
Conf:
Pred:
Pred: CCCEEEEEECCEEEECCCCEEEEEEEEEECCEEEECCCEE
AA: PSGFTVAAGNGSVTTWTGNWGTSWGVKGFGGVIGVTDELQ
730 740 750 760
Conf:
Pred:
Pred: EEEECEEECCEEEEECCEEEEECCCEEEEEEEECCCEEEE
AA: YDVGTGLTEELIFGLGGKTSRVDTRLDLFMREGAFNSFAE
770 780 790 800
Conf:
Pred:
Pred: EEEEEEEEEEECCCEEEEECEEEEEEECCCCEEEEEECCE
AA: RAQVEMFKTTTTAGDILSRQQTATLTANDATRVYGDVNPT
810 820 830 840

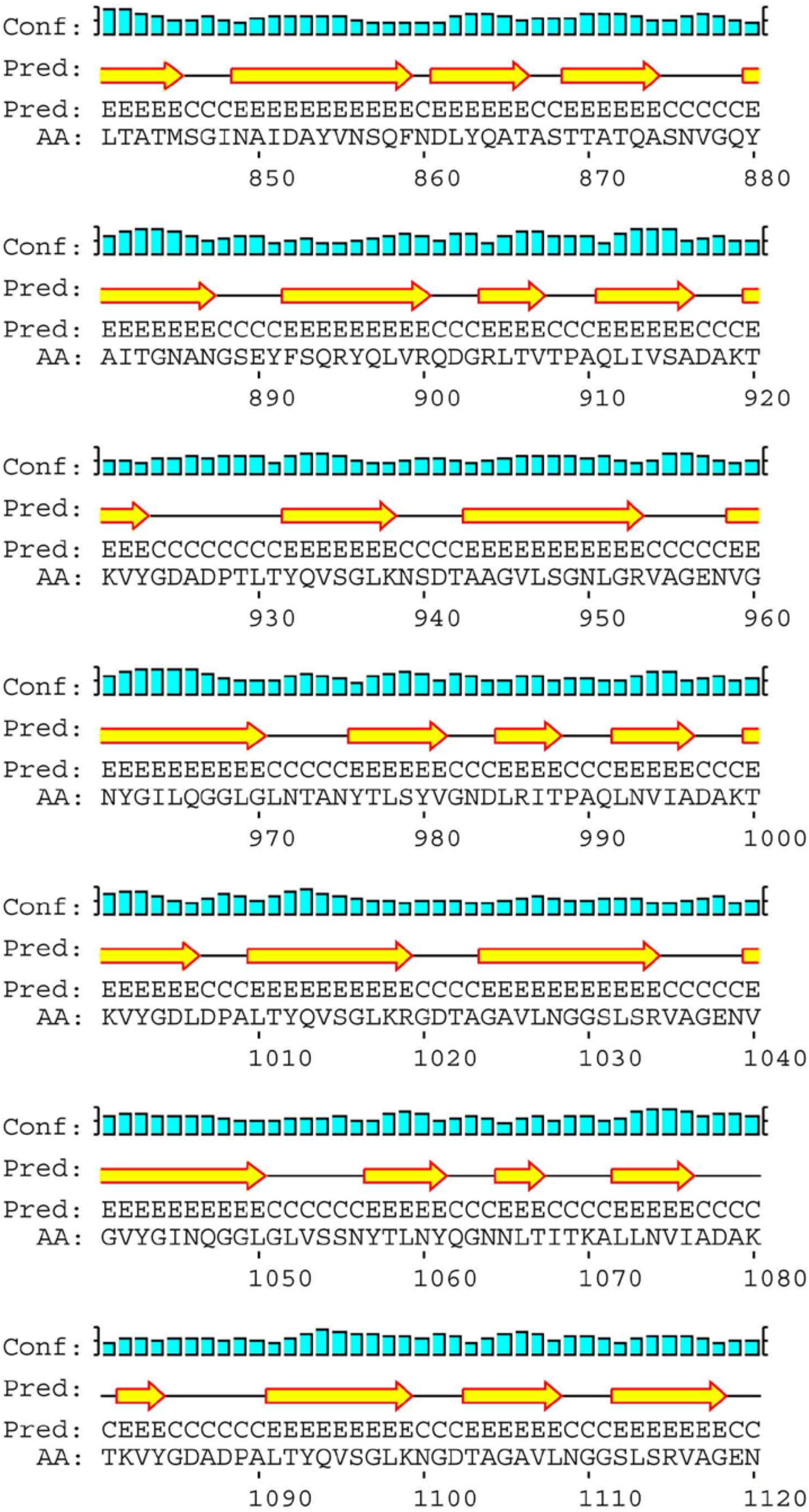
Conf:
Pred:
Pred: EEEEECCCEEEEEEEEEEECEEEEEECCEEEEEECCCCCE
AA: LTATMSGINAIDAYVNSQFNDLYQATASTTATQASNVGQY
850 860 870 880
Conf:
Pred:
Pred: EEEEEEECCCCEEEEEEEEECCCEEEECCCEEEEEECCCE
AA: AITGNANGSEYFSQRYQLVRQDGRLTVTPAQLIVSADAKT
890 900 910 920
Conf:
Pred:
Pred: EEECCCCCCCCEEEEEEECCCCEEEEEEEEEEECCCCCEE
AA: KVYGDADPTLTYQVSGLKNSDTAAGVLSGNLGRVAGENVG
930 940 950 960
Conf:
Pred:
Pred: EEEEEEEEEECCCCCEEEEEECCCEEEECCCEEEEECCCE
AA: NYGILQGGLGLNTANYTLSYVGNDLRITPAQLNVIADAKT
970 980 990 1000
Conf:
Pred:
Pred: EEEEEECCCEEEEEEEEEECCCCEEEEEEEEEEECCCCCE
AA: KVYGDLDPALTYQVSGLKRGDTAGAVLNGGSLSRVAGENV
1010 1020 1030 1040
Conf:
Pred:
Pred: EEEEEEEEEECCCCCCEEEEECCCEEECCCCEEEEECCCC
AA: GVYGINQGGLGLVSSNYTLNYQGNNLTITKALLNVIADAK
1050 1060 1070 1080
Conf:
Pred:
Pred: CEEECCCCCCEEEEEEEEECCCEEEEEECCCEEEEEEECC
AA: TKVYGDADPALTYQVSGLKNGDTAGAVLNGGSLSRVAGEN
1090 1100 1110 1120

```
Conf:
Pred:
Pred: EEEEEEEECCCCCCCEEEEEEECCCCEEECCCEEEEEECC
  AA: VGVYGINQGGLGLLSANYDLSYQGNNLTITKALLNVIADA
                1130      1140      1150      1160

Conf:
Pred:
Pred: CCEEECCEEEEECCEEEEEECCCEEEEEEEECCCCCCCCC
  AA: KTKVYGDADPSLTYQVSGLKNGDTAGSILTGGLNRAAGEN
                1170      1180      1190      1200

Conf:
Pred:
Pred: EEEEEECCCEEEECCCCCEEECCCCCCCCCCCEEEEEECC
  AA: VGVYGINQGDLALNSGNYDLSYQGNNLTITKALLNVIADA
                1210      1220      1230      1240

Conf:
Pred:
Pred: CEEEECCCCCCEEEEEEEEEECCEEEEEECCCEEEEECCC
  AA: KTKVYGDADPSLTYQVSGLKNGDTAGAVLNGGGLVRVSGE
                1250      1260      1270      1280

Conf:
Pred:
Pred: CCCEEEEEECCCEECCCEEEEEECCCCEECCCCEEEEEEC
  AA: NVGNYAIQQGGLGLVSGNYDLAYQGNNLTITKALLNVIAD
                1290      1300      1310      1320

Conf:
Pred:
Pred: CCCEEECCCCCCCEEEEEEECCCCCEEEEEECCCCCCCCE
  AA: AKTKVYGDADPSLTYQVSGLKNGDSAGSILTGGLNRAAGE
                1330      1340      1350      1360

Conf:
Pred:
Pred: EEEEEEEEECCEECCCCEEEEEECCCEEEECCCCEEEEEC
  AA: NVGVYGINQGDLALNSGNYDLSYQGNNLTITKALLNVIAD
                1370      1380      1390      1400
```

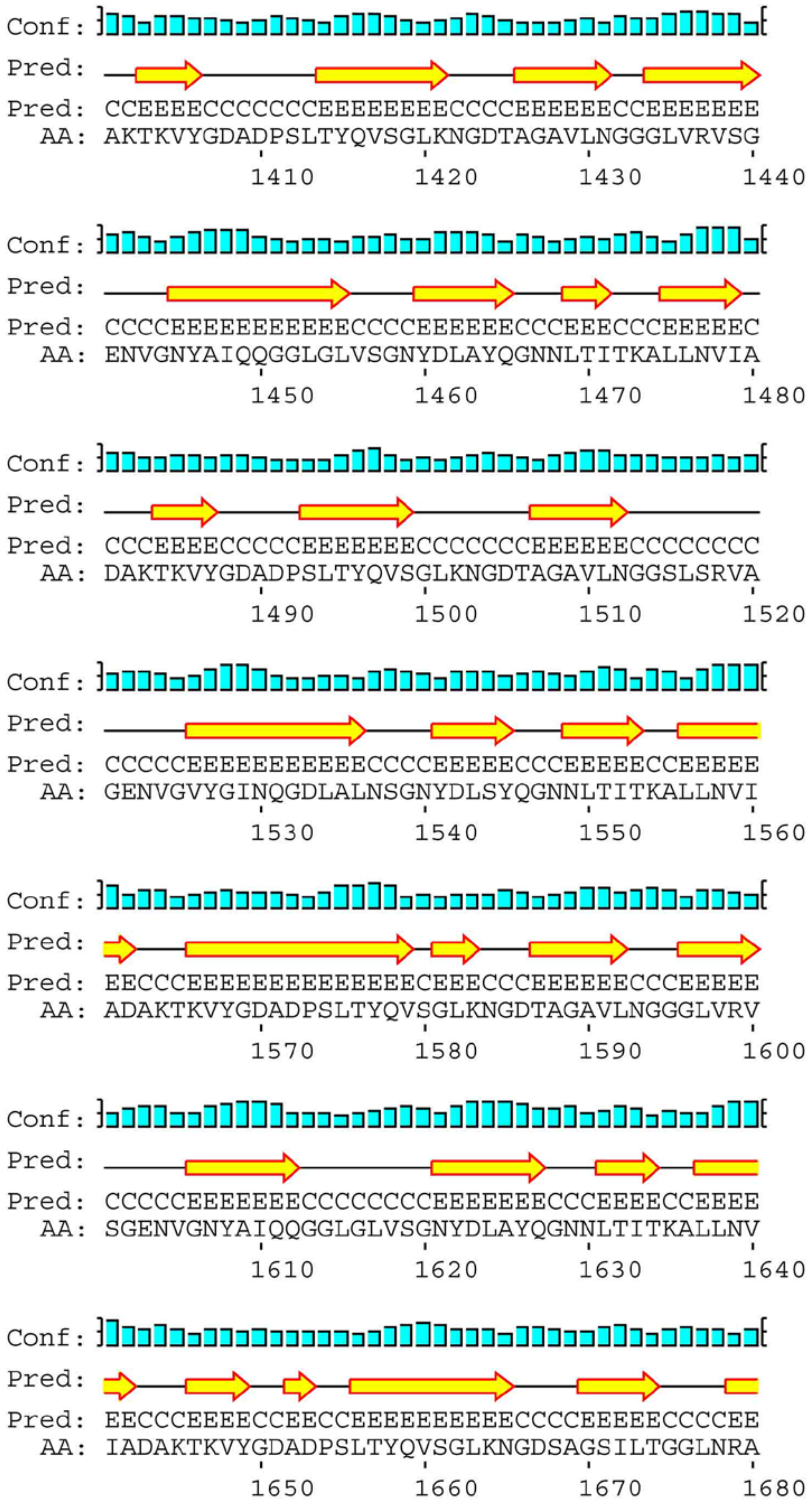

Conf:
Pred:
Pred: CCEEEECCCCCCCEEEEEEEECCCCEEEEEECCEEEEEEE
AA: AKTKVYGDADPSLTYQVSGLKNGDTAGAVLNGGGLVRVSG
1410 1420 1430 1440
Conf:
Pred:
Pred: CCCCEEEEEEEEEEECCCCEEEEEECCCEEECCCEEEEEC
AA: ENVGNYAIQQGGLGLVSGNYDLAYQGNNLTITKALLNVIA
1450 1460 1470 1480
Conf:
Pred:
Pred: CCCEEEECCCCCEEEEEEECCCCCCCEEEEEECCCCCCCC
AA: DAKTKVYGDADPSLTYQVSGLKNGDTAGAVLNGGSLSRVA
1490 1500 1510 1520
Conf:
Pred:
Pred: CCCCCEEEEEEEEEEECCCCEEEEECCCEEEEECCEEEEE
AA: GENVGVYGINQGDLALNSGNYDLSYQGNNLTITKALLNVI
1530 1540 1550 1560
Conf:
Pred:
Pred: EECCCEEEEEEEEEEEEEECEEECCCEEEEEECCCEEEEE
AA: ADAKTKVYGDADPSLTYQVSGLKNGDTAGAVLNGGGLVRV
1570 1580 1590 1600
Conf:
Pred:
Pred: CCCCCEEEEEEECCCCCCCCEEEEEEECCCEEEECCEEEE
AA: SGENVGNYAIQQGGLGLVSGNYDLAYQGNNLTITKALLNV
1610 1620 1630 1640
Conf:
Pred:
Pred: EECCCEEEECCEECCEEEEEEEEEECCCCEEEEECCCCEE
AA: IADAKTKVYGDADPSLTYQVSGLKNGDSAGSILTGGLNRA
1650 1660 1670 1680

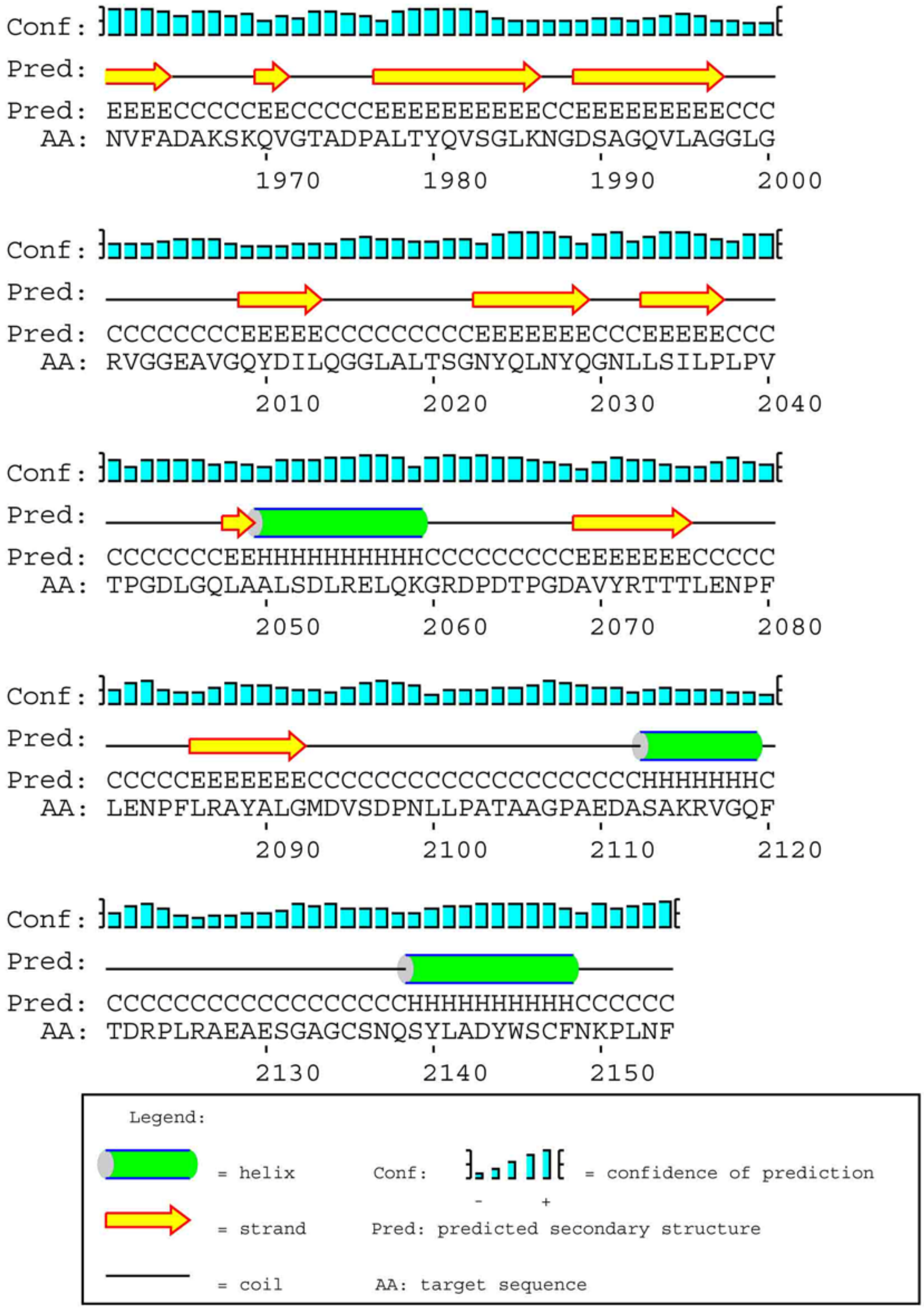
Conf:
Pred:
Pred: EEEECCCCCEECCCCCEEEEEEEEEECCEEEEEEEEEECCC
AA: NVFADAKSKQVGTADPALTYQVSGLKNGDSAGQVLAGGLG
1970 1980 1990 2000
Conf:
Pred:
Pred: CCCCCCCCEEEEECCCCCCCCCEEEEEEECCCEEEEEECCC
AA: RVGGEAVGQYDILQGGLALTSGNYQLNYQGNLLSILPLPV
2010 2020 2030 2040
Conf:
Pred:
Pred: CCCCCCCEEHHHHHHHHHHCCCCCCCCCEEEEEEECCCCC
AA: TPGDLGQLAALSDLRELQKGRDPDTPGDAVYRTTTLENPF
2050 2060 2070 2080
Conf:
Pred:
Pred: CCCCCEEEEEEECCCCCCCCCCCCCCCCCCCHHHHHHHC
AA: LENPFLRAYALGMDVSDPNLLPATAAGPAEDASAKRVGQF
2090 2100 2110 2120
Conf:
Pred:
Pred: CCCCCCCCCCCCCCCCCCHHHHHHHHHHCCCCCC
AA: TDRPLRAEAESGAGCSNQSYLADYWSCFNKPLNF
2130 2140 2150
Legend:
= helix
= strand
= coil
Conf:
= confidence of prediction
- +
Pred: predicted secondary structure
AA: target sequence

Supp. Fig. 1

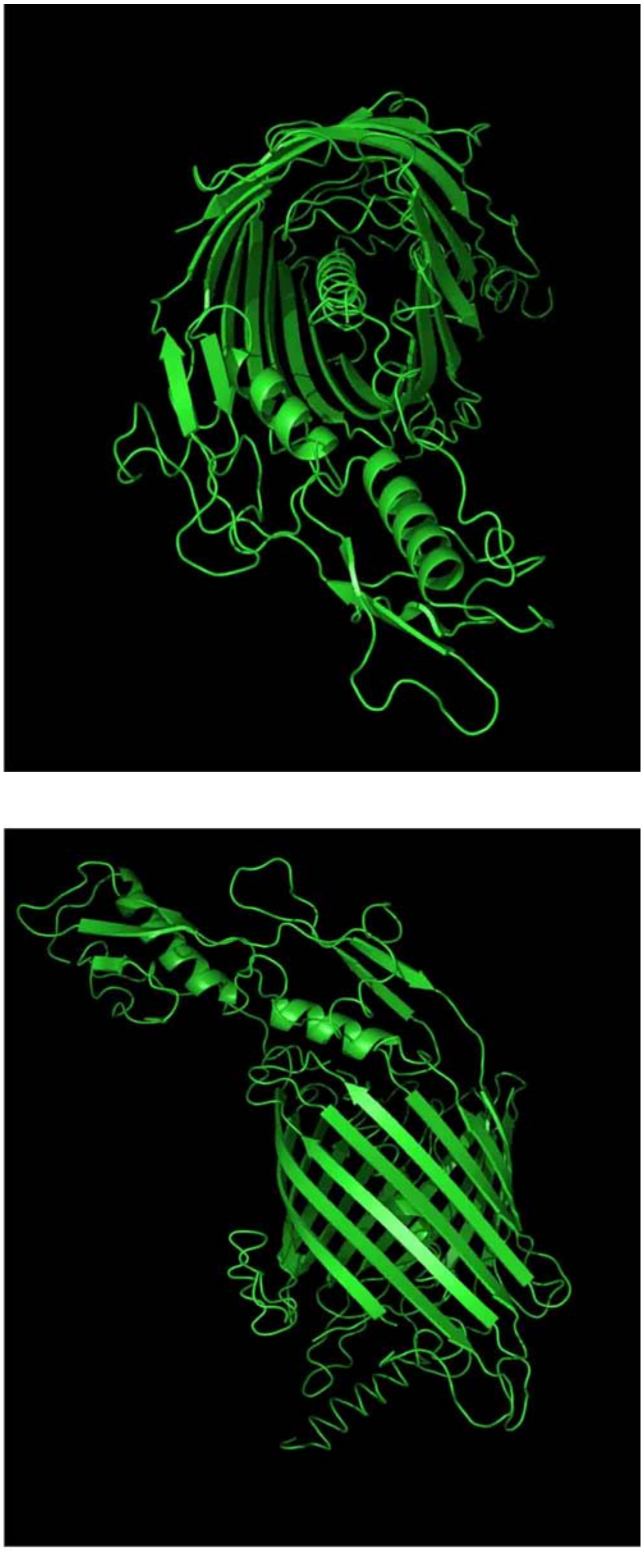

Supp. Fig. 2

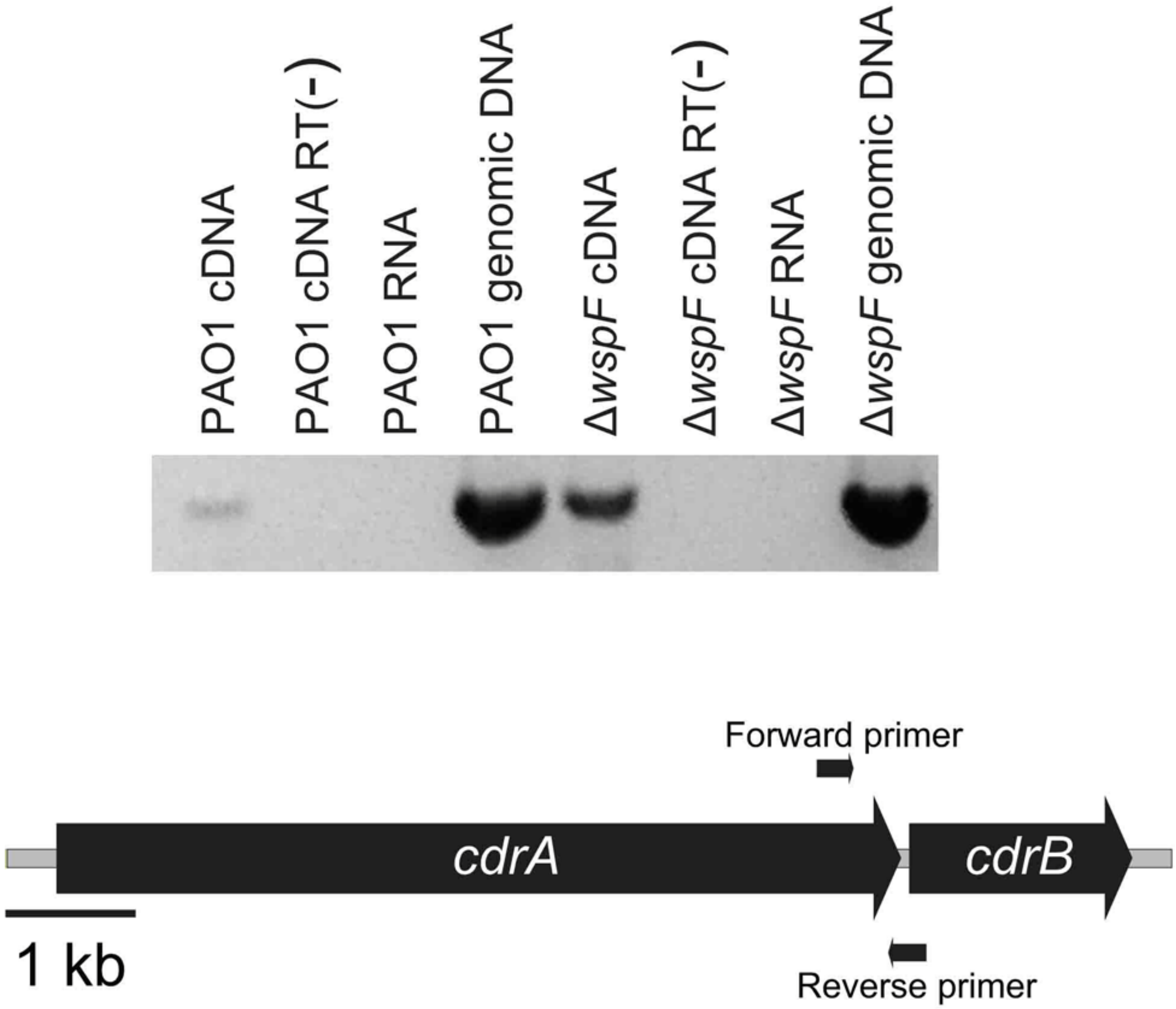
PAO1 cDNA
PAO1 cDNA RT(-)
PAO1 RNA
PAO1 genomic DNA
ΔwspF cDNA
ΔwspF cDNA RT(-)
ΔwspF RNA
ΔwspF genomic DNA
Forward primer
cdrA
cdrB
1 kb
Reverse primer

Supp. Fig. 3

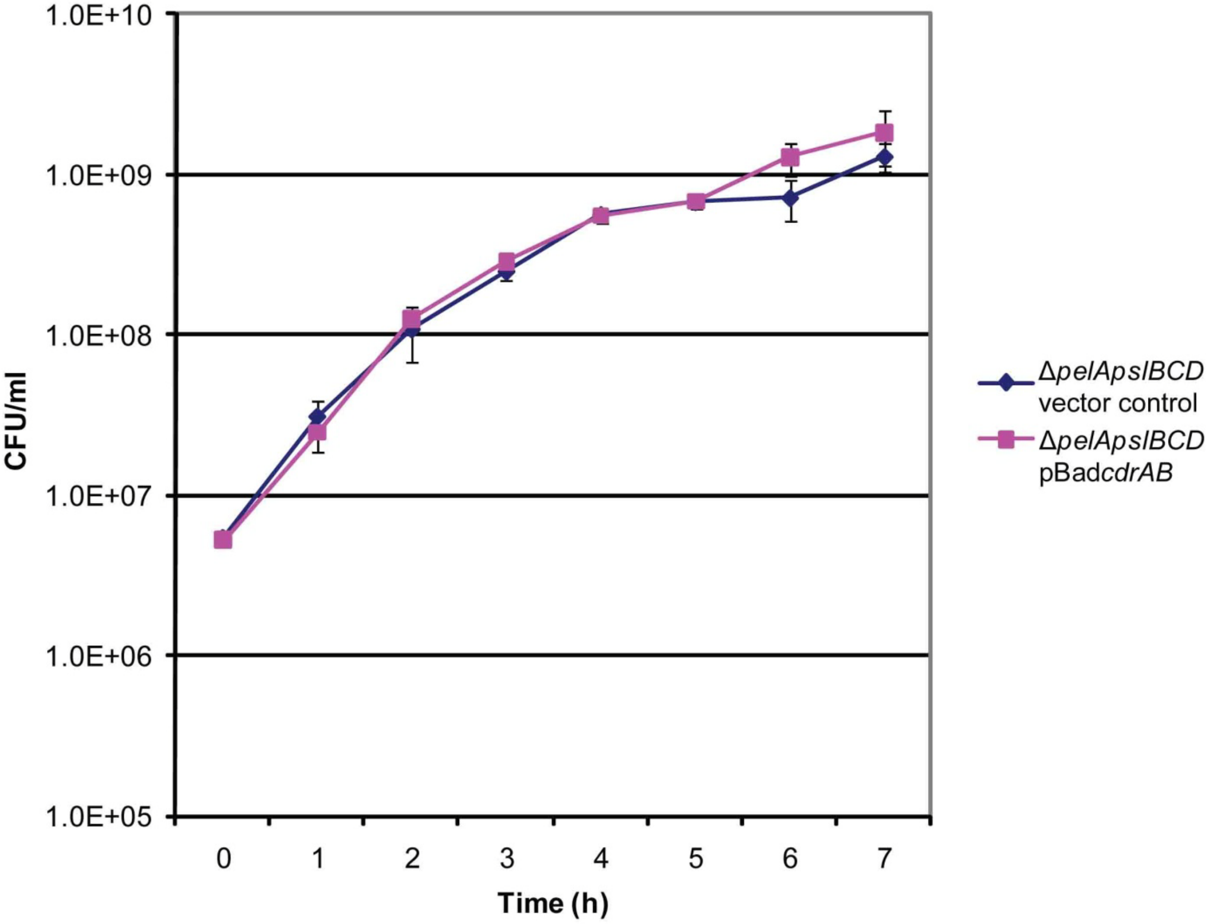
1.0E+10
1.0E+09
1.0E+08
1.0E+07
1.0E+06
1.0E+05
CFU/ml
0
1
2
3
4
5
6
7
Time (h)
ΔpelApslBCD vector control
ΔpelApslBCD pBadcdrAB

Supp. Fig. 4

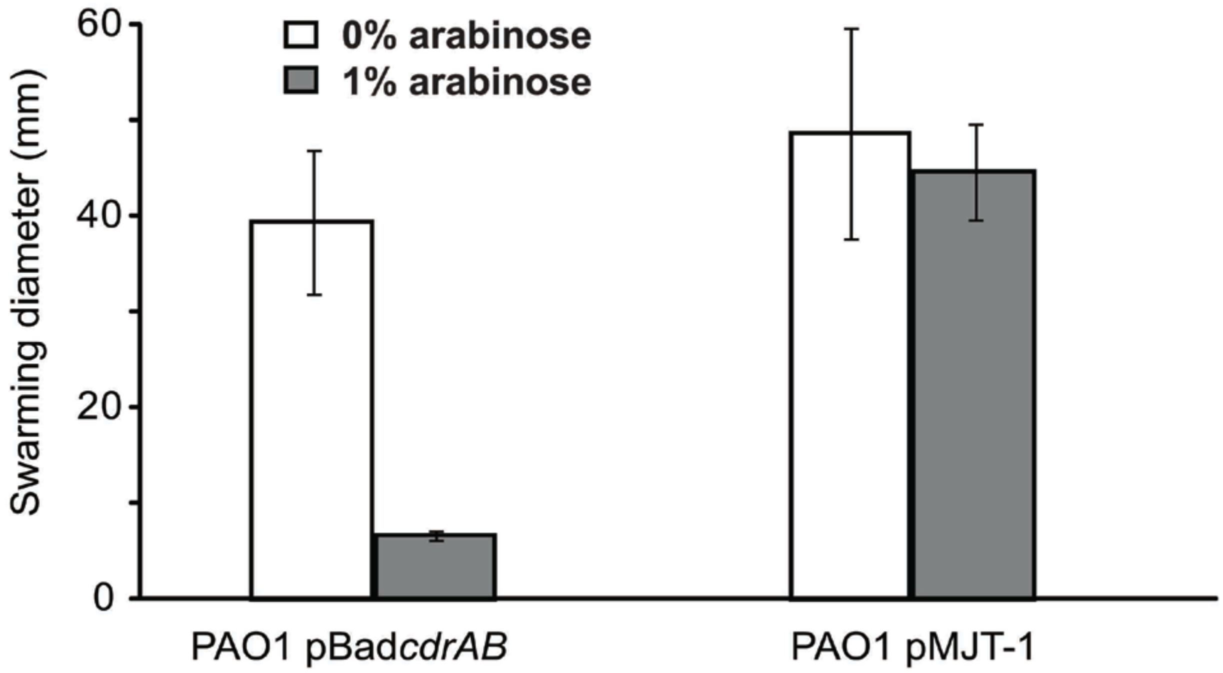
60
40
20
0
Swarming diameter (mm)
0% arabinose
1% arabinose
PAO1 pBadcdrAB
PAO1 pMJT-1

Supp. Fig. 5

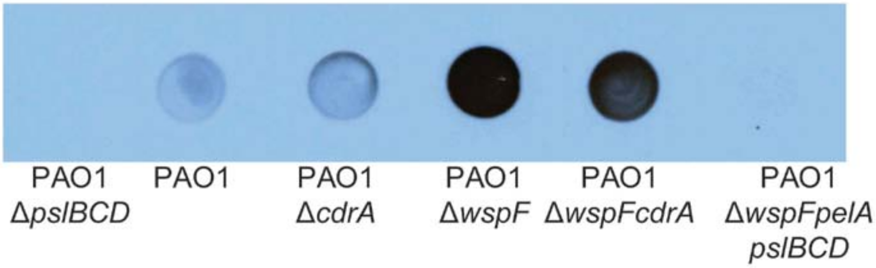

Supp. Fig. 6

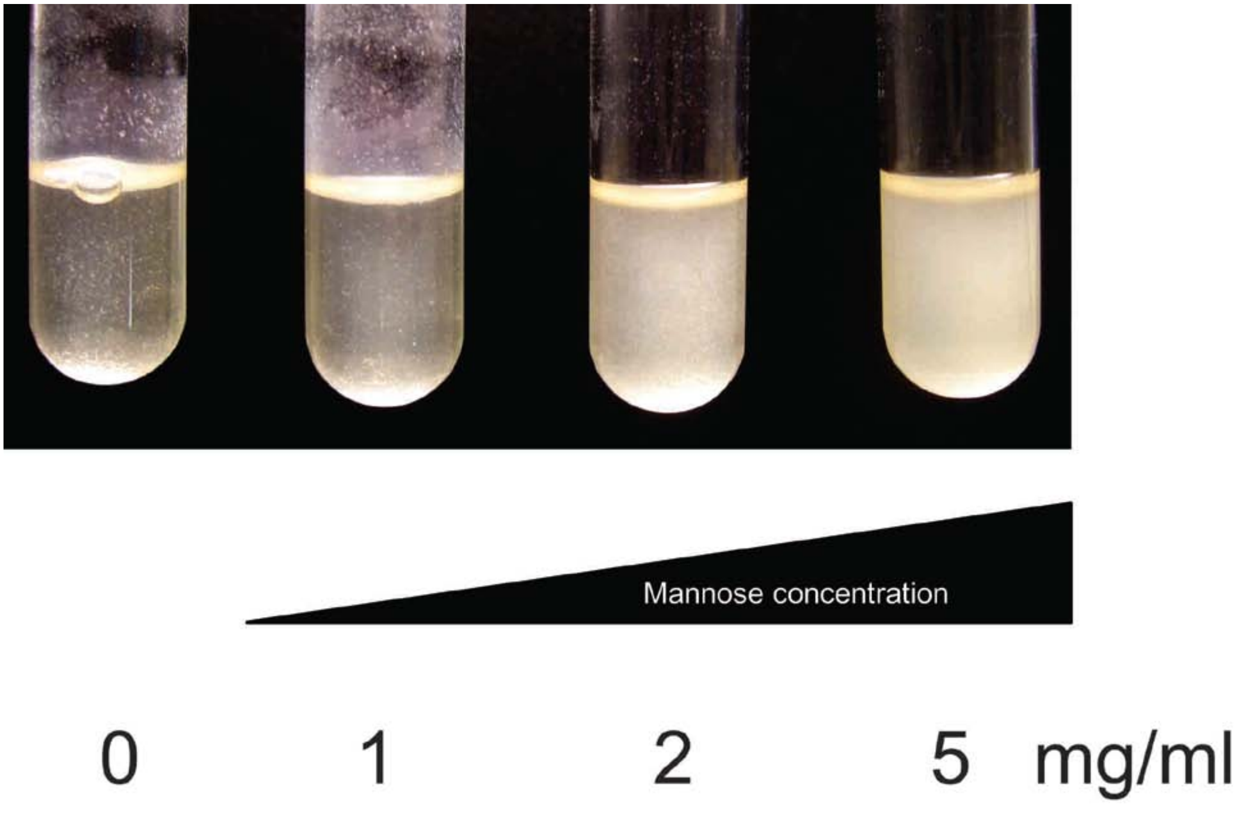

Supp. Fig. 7

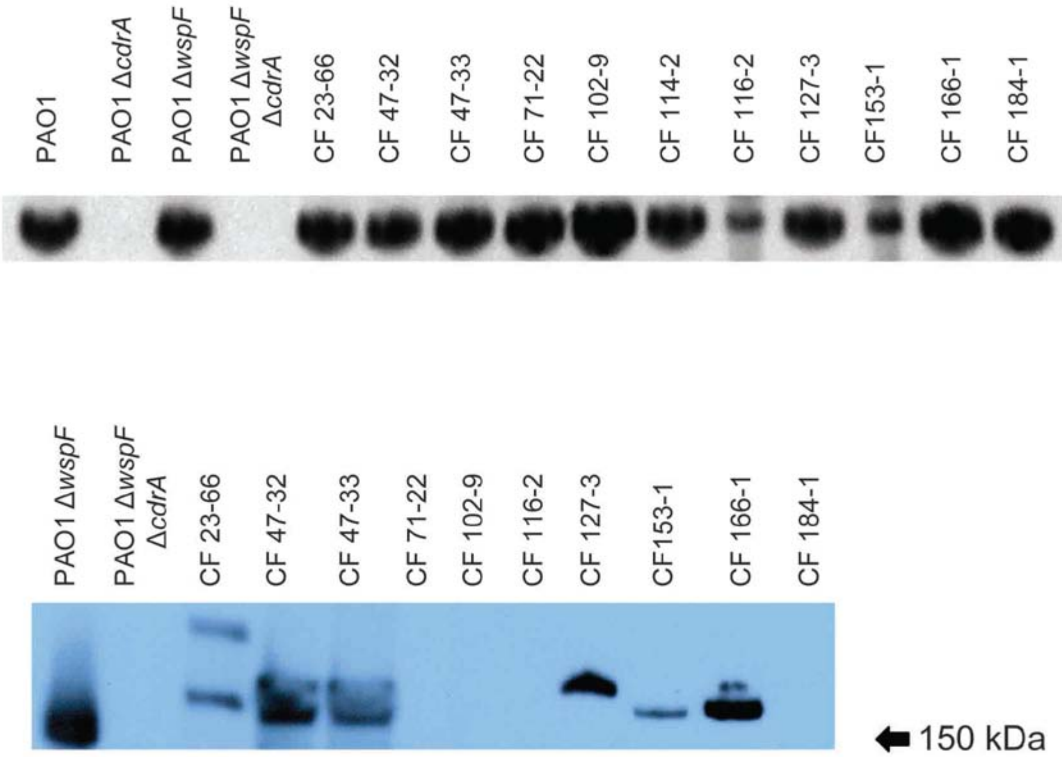
PAO1
PAO1 ΔcdrA
PAO1 ΔwspF
PAO1 ΔwspF ΔcdrA
CF 23-66
CF 47-32
CF 47-33
CF 71-22
CF 102-9
CF 114-2
CF 116-2
CF 127-3
CF153-1
CF 166-1
CF 184-1
PAO1 ΔwspF
PAO1 ΔwspF ΔcdrA
CF 23-66
CF 47-32
CF 47-33
CF 71-22
CF 102-9
CF 116-2
CF 127-3
CF153-1
CF 166-1
CF 184-1
150 kDa

Supp. Fig. 8

Supp. Table 1

Primer	Sequence
**pEX4625 (Δ*cdrA*)**	
BB15_5'RinX4625	GCAGCTCCAGTAGTCGGCCAGCTGGCTATCCGGACGGACCATGAA
BB16_5'RouthindIIIX4625	AAGCTTCAGGAGTTCGCCAAGCGGCAG
BB17_3'LinX4625	ATGGTCCGTCCGGATAGCCAGCTGGCCGACTACTGGAGCTGC
BB18_3'LoutxbaIX4625	TCTAGACCATGATCTGCAGGGTATCGC
**pBAD*cdrAB***	
BB08	TATCTAGAATAGGGAGATTTTCATGGTCCGTCCG
BB09	TAGAATTCTCAGAAGCGCGCCACCACGTTGAACAGG
**pBB022 (*cdrA* amino acids 770-936)**	
BB66_aa770HindIII	ATAAAGCTTGAACTGATCTTCGGCCTCGGCGG
BB67_aa936NotI	ATAGCGGCCGCGCCACTGACCTGGTAGGTGA
**pBAD*cdrA***	
BB08	TATCTAGAATAGGGAGATTTTCATGGTCCGTCCG
BB72	TAGAATTCGAGACGATAGTGCGCATTGC
**pBAD*cdrB* (Δ*cdrA* DNA template for PCR)**	
BB08	TATCTAGAATAGGGAGATTTTCATGGTCCGTCCG
BB09	TAGAATTCTCAGAAGCGCGCCACCACGTTGAACAGG
**Detection of *cdrA* in clinical strains**	
BB19	CTGGTAGTTCAGGGTGTAATTGCT
BB20	AGGGAGCGTTCAACAGCTTC
**Operon confirmation**	
BB77	CTGCGGGGCTTGAAGCGTTCAT
BB78	CCTGGTGCTGACCAGTGGCA

Supplementary Figure legends

Supplementary Figure 1- The predicted secondary structure of CdrA by Psi Pred. software.

Supplementary Figure 2- The predicted tertiary structure of CdrB. This prediction was achieved through use of I=TASSER software.

Supplementary Figure 3. *cdrA* and *cdrB* are co-transcribed. (Top) RT-PCR products from mRNA derived from PAO1 and wspF strains. The lanes indicated by (PAO1 cDNA and wspF cDNA) are positive for a cDNA product derived from a single transcript, indicating that cdrAB are co-transcribed. The lanes marked cDNA (RT-) represent a negative control of reactions lacking reverse transcriptase, while lanes marked RNA are negative controls showing RNA only loaded. The lanes marked genomic DNA are amplification of the PCR product using a DNA template. (Bottom) A diagram depicting the genetic organization of the *cdrAB operon*.

Supplementary Figure 4-Growth curves showing the effect of *cdrAB* overexpression.

Supplementary Figure 5.- The effects of *cdrAB* expression on swarming motility.

Supplementary Figure 6- Dot blots showing relative Psl levels in select strains using anti-Psl antisera.

Supplementary Figure 7. Figure showing the effects of increasing exogenous mannose levels on *cdrAB*-mediated autoaggregation. Mannose disrupted autoaggregation, indicating it can bind to CdrA.

Supplemental Figure 8- Clinical isolates have the cdrA gene (top) and most express the protein (bottom). (Top) An agarose gel showing PCR products generated using primers specific for DNA within the cdrA gene, using different clinical isolates as sources for template DNA. (Bottom) A western blot showing CdrA-reactive proteins produced by different clinical strains.
